# Herpes simplex virus 1 protein pUL21 alters ceramide metabolism by activating the interorganelle transport protein CERT

**DOI:** 10.1016/j.jbc.2022.102589

**Published:** 2022-10-13

**Authors:** Tomasz H. Benedyk, Viv Connor, Eve R. Caroe, Maria Shamin, Dmitri I. Svergun, Janet E. Deane, Cy M. Jeffries, Colin M. Crump, Stephen C. Graham

**Affiliations:** 1 Department of Pathology, University of Cambridge, Cambridge, UK; 2 Cambridge Institute for Medical Research, University of Cambridge, Cambridge, UK; 3 European Molecular Biology Laboratory (EMBL) Hamburg Site, Hamburg, Germany

**Keywords:** virus–host interactions, ceramide transport, herpesvirus, lipid metabolism, protein phosphatases, BAC, bacterial artificial chromosome, Cer, ceramide, DMEM, dulbecco’s modified eagle's medium, DMSO, dimethyl sulfoxide, ER, endoplasmic reticulum, GST, glutathione-*S*-transferase, HEK293T, human embryonic kidney 293T cell line, hpi, hours postinfection, HPTLC, high-performance TLC, HSV, herpes simplex virus, ITC, isothermal titration calorimetry, MOI, multiplicity of infection, MR, middle region, NEC, nuclear egress complex, PC, phosphatidylcholine, PDB, protein data bank, PH, pleckstrin homology, PP1, protein phosphatase 1, RT, room temperature, SAXS, small-angle X-ray scattering, SEC–MALS, size-exclusion chromatography with inline multiangle light scattering, SM, sphingomyelin, Sph, sphingosine, START, steroidogenic acute regulatory–related lipid transfer, TCEP, Tris(2-carboxyethyl)phosphine, TGN, *trans*-golgi network

## Abstract

Herpes simplex virus (HSV)-1 dramatically alters the architecture and protein composition of cellular membranes during infection, but its effects upon membrane lipid composition remain unclear. HSV-1 pUL21 is a virus-encoded protein phosphatase adaptor that promotes dephosphorylation of multiple cellular and virus proteins, including the cellular ceramide (Cer) transport protein CERT. CERT mediates nonvesicular Cer transport from the endoplasmic reticulum to the *trans*-Golgi network, whereupon Cer is converted to sphingomyelin (SM) and other sphingolipids that play important roles in cellular proliferation, signaling, and membrane trafficking. Here, we use click chemistry to profile the kinetics of sphingolipid metabolism, showing that pUL21-mediated dephosphorylation activates CERT and accelerates Cer-to-SM conversion. Purified pUL21 and full-length CERT interact with submicromolar affinity, and we solve the solution structure of the pUL21 C-terminal domain in complex with the CERT Pleckstrin homology and steroidogenic acute regulatory–related lipid transfer domains using small-angle X-ray scattering. We identify a single amino acid mutation on the surface of pUL21 that disrupts CERT binding *in vitro* and in cultured cells. This residue is highly conserved across the genus *Simplexvirus*. In addition, we identify a pUL21 residue essential for binding to HSV-1 pUL16. Sphingolipid profiling demonstrates that Cer-to-SM conversion is severely diminished in the context of HSV-1 infection, a defect that is compounded when infecting with a virus encoding the mutated form of pUL21 that lacks the ability to activate CERT. However, virus replication and spread in cultured keratinocytes or epithelial cells is not significantly altered when pUL21-mediated CERT dephosphorylation is abolished. Collectively, we demonstrate that HSV-1 modifies sphingolipid metabolism *via* specific protein–protein interactions.

Herpes simplex virus (HSV)-1 is a human pathogen that is estimated to infect the majority of the human population, causing a lifelong latent infection (1). Latent HSV-1 resides in sensory neurons or sympathetic neurons, migrating to the periphery in periodic reactivation events throughout the lifetime of the host. In order to sustain acute (lytic) infection, HSV-1 drastically modifies infected cells (2, 3, 4, 5). In particular, the virus extensively remodels the composition and architecture of cellular membranes to facilitate virus assembly and spread. Nascent capsids leave the nucleus *via* sequential envelopment and de-envelopment at the inner and outer nuclear membranes (6). These cytosolic capsids acquire a proteinaceous layer termed “tegument” and bud into the lumen of post-Golgi membranes that are studded with viral glycoproteins (so called “secondary envelopment”) (7). The resultant virus-containing vesicles are transported to cell contact sites where they release the mature virus particles to disseminate the infection (8).

HSV-1 pUL21 is a tegument protein that is conserved in all alphaherpesviruses (9). This multifunctional protein and its homologs are known to interact with multiple cellular and viral partners, including pUL16 (10, 11), pUL11 (12), gE (12), tubulin (13), and Roadblock-1 (14), and it has been implicated in a number of important processes including capsid nuclear egress (15), viral cell-to-cell spread (12, 16), and retrograde transport along axons to the neuronal cell bodies where latency is established (14, 17). Mutant HSV-1 lacking pUL21 expression exhibits a 10-fold to 100-fold replication defect and severely impaired cell-to-cell spread, both of which can be at least partially ascribed to the phosphomodulatory role of pUL21 as a protein phosphatase 1 (PP1) adaptor (18). PP1 is a highly active and abundant cellular phosphatase (19), and pUL21 recruits PP1 to promote dephosphorylation of multiple substrates, including the viral protein pUL31 that is implicated in viral nuclear egress (18, 20) and the cellular protein CERT that regulates sphingomyelin (SM) metabolism (18).

The cytoplasmic ceramide (Cer) transport protein CERT (a.k.a. Goodpasture’s antigen-binding protein, GPBP, encoded by the gene *COL4A3BP*) mediates the nonvesicular trafficking of Cer from the endoplasmic reticulum (ER) to the *trans*-Golgi network (TGN) and, in doing so, defines the rate of SM synthesis (21, 22, 23). CERT contains two well-folded globular domains: an N-terminal Pleckstrin homology (PH) domain that mediates its interaction with TGN membranes by binding phosphatidylinositol 4-phosphate and a C-terminal steroidogenic acute regulatory–related lipid transfer (START) domain that directly binds Cer to mediate its transfer (23). Crystal structures of both domains have been solved (24, 25). The “middle region” (MR) that connects the PH and START domains of CERT does not adopt a globular fold, instead containing a coiled-coil region that is likely to mediate CERT self-association (26), a “two phenylalanines in an acidic tract” (FFAT) motif that recruits CERT to the ER membranes *via* binding proteins VAPA and VAPB (27), and a serine-rich motif, hyperphosphorylation of which represses CERT activity (28). In cultured HeLa cells, the vast majority of CERT is in an inactive hyperphosphorylated state (CERT^P^), available to be mobilized *via* dephosphorylation to increase the rate of ER-to-TGN Cer transport in response to stimuli such as SM depletion (28).

Cer and sphingolipids like SM are essential for mammalian cell growth, and they play important roles in cell signaling, apoptosis, and membrane trafficking (29). Furthermore, sphingolipids and cholesterol participate in the formation of membrane microdomains (including “lipid rafts”) that compartmentalize the lipid and protein composition of cellular membranes (30). This compartmentalization is especially important in highly polarized cells such as neurons (31). Although sphingolipids are crucial to host cell biology, relatively little is known about the interaction between pathogens and cellular sphingolipid metabolism. The bacteria *Chlamydia trachomatis* is known to directly recruit CERT to bacteria-containing intracellular inclusions, thereby increasing the abundance of SM in the inclusion membrane (32), and CERT activity is necessary for the biosynthesis of double-membrane vesicles that serve as the sites of hepatitis C virus and polio virus replication (33). However, to the best of our knowledge, the binding of CERT to HSV-1 pUL21 (18) is the only known example of a direct interaction between CERT and a virus protein.

Previous studies have shown that HSV-1 infection increases Cer synthesis (34) and that depletion of sphingomyelinase causes a >30-fold decrease in HSV-1 replication (35). Sphingomyelinase treatment of cultured epithelial cells has only a very modest effect upon HSV-1 entry (36), suggesting that SM is not absolutely required for virus infection. In contrast, recent studies in macrophages demonstrated that acid ceramidase activity is potently antiviral, helping sequester incoming virus particles by promoting their neutralizing association with sphingosine (Sph)-rich intraluminal vesicles within endocytic multivesicular bodies (37). Furthermore, siRNA depletion of CERT has been shown to promote secretion of usually cell-associated HSV-1 virions (38). It is therefore increasingly clear that cellular sphingolipid metabolism is important for HSV-1 biology and that further studies are required to obtain a full picture of how specific sphingolipids support and/or prevent HSV-1 infection.

We sought to define the functional consequences of the pUL21–CERT interaction for HSV-1 infection. Using click chemistry, we demonstrated that pUL21-mediated CERT dephosphorylation increases the rate of Cer to SM conversion in cultured cells. We characterized the solution structure of the pUL21 C-terminal domain in complex with the PH and START domains of CERT, identifying a specific pUL21 amino acid required for the interaction. Functional characterization of mutant HSV-1 encoding this mutated form of pUL21 confirmed that pUL21-mediated CERT dephosphorylation alters the rate of SM synthesis in infected cells. Furthermore, we observed a dramatic increase in the rate of Cer accumulation in infected cells, which was only partly counteracted by pUL21-stimulated CERT activity, but this increase in Cer abundance does not significantly alter virus replication or spread in cultured keratinocytes or epithelial cells.

## Results

**Figure 1:**
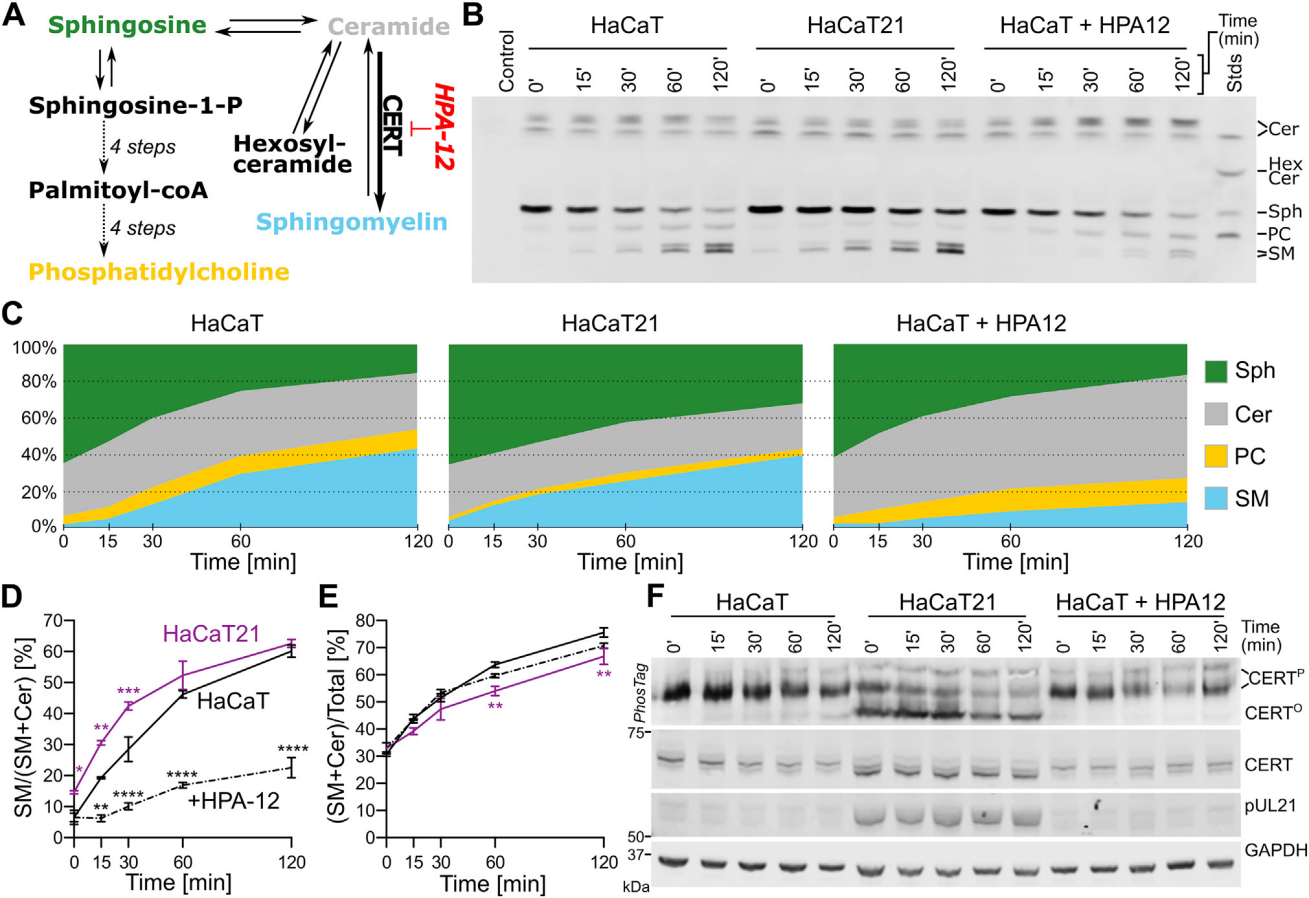
**Herpes simplex virus 1 (HSV-1) pUL21 alters the rate of ceramide (Cer) to sphingomyelin (SM) conversion in cultured cells.***A*, simplified schematic diagram of sphingolipid biosynthetic pathways that lead from sphingosine (Sph) to SM and glycosphingolipids (hexosylceramide; HexCer), or to phosphatidylcholine (PC) *via* a “salvage” pathway. In this salvage pathway, Sph-1-phosphate is converted to *trans*-2-hexadecenal, then *trans*-2-hexadecenoic acid and *trans*-2-hexadecenoyl-coA before being converted to palmitoyl-coA (112), which is in turn conjugated to glycerol-3-phosphate to form lysophosphatidic acid before being converted to phosphatidic acid, diacylglycerol, and then PC (113). Cer to SM conversion is accelerated by the transport protein CERT, which is selectively inhibited by HPA-12. *B*, rate of Sph conversion to Cer, SM, or PC was measured in HaCaT cells, untreated or treated with 1 μM HPA-12, and in HaCaT cells stably expressing pUL21 (HaCaT21). Cells were incubated with 5 μM “clickable” alkyne-Sph (pulse) for 5 min and harvested for lipid extraction either immediately (0 min) or at the indicated times (chase). Extracted lipids were bioconjugated to 3-azido-7-hydroxycoumarin, separated by HPTLC and detected using UV light. Separated lipids were identified using clickable standards (Stds) and previous literature (42). Data are from one representative experiment of two independent repeats. *C*, quantitation of the lipid intensities from (*B*) as determined by densitometry and represented as percentage fraction of total signal. *D*, rate of SM synthesis expressed as its fraction in the cumulative signal for SM and Cer. *E*, the ratio of alkyne-Sph incorporated into either Cer or SM as a proportion of total alkyne-lipid signal, representing the influx of Sph into the SM biosynthetic pathway. For *D* and *E*, the data represent two independent experiments (mean ± SD). Data points are labeled if significantly different to parental HaCaT cells: ∗*p* < 0.05; ∗∗*p* < 0.01; ∗∗∗*p* < 0.001; and ∗∗∗∗*p* < 0.0001 (two-way ANOVA with Dunnett’s multiple comparisons test). *F*, the resolubilized proteins precipitated during lipid extraction were analyzed by SDS-PAGE and immunoblotting using the antibodies listed. Where indicated, the gel was supplemented with PhosTag reagent to retard the migration of phosphorylated proteins, thus enhancing the separation of CERT that is hypophosphorylated (CERT^O^) or hyperphosphorylated (CERT^P^). GAPDH serves as a loading control.

Human cells typically maintain lipidome homeostasis *via* robust feedback and control mechanisms, which respond to perturbations *via* local activation of specific signaling pathways (39, 40). We hypothesized that pUL21, expressed at late points of infection (3), would likely alter the rate of sphingolipid synthesis and thereby affect lipid-mediated signaling events, rather than altering the overall steady-state lipid composition of infected cells. Synthetic lipids with alkyne-containing acyl chains are efficiently processed by mammalian lipid-modifying enzymes and represent powerful tools to probe lipid metabolism (41). The impact of pUL21-directed CERT dephosphorylation on sphingolipid metabolism was therefore probed using a clickable analog of Sph, alkyne-Sph, to monitor the rate of SM biogenesis in immortalized human keratinocyte (HaCaT) cells. Exogenous Sph is efficiently incorporated into cellular metabolic pathways, being rapidly converted into Cer and then SM or hexosylceramides like glucosylceramide or galactosylceramide (42). It is also converted into phosphatidylcholine (PC) *via* a so-called “salvage” pathway (Fig. 1*A*) that directs Sph to palmitoyl-CoA, which serves as substrate for reacetylation of lysophosphatidylcholine (43).

To monitor Sph metabolism, HaCaT cells, either parental or stably expressing pUL21 (HaCaT21), were incubated with alkyne-Sph for 5 min (pulse), and the rate of alkyne-Sph incorporation into the competing metabolic pathways was monitored for 2 h (chase) by high-performance TLC (HPTLC) separation and detection of lipids conjugated to coumarin-azide *via* a “click” reaction (Fig. 1, *B* and *C*). Alkyne-Sph was very efficiently converted to alkyne-Cer in both cell types, the levels of alkyne-Cer remaining relatively stable throughout the chase, and for both cell types, synthesis of alkyne-PC and alkyne-SM was observed but not synthesis of alkyne-hexosylceramide. HaCaT21 cells exhibited significantly reduced rates of alkyne-Sph conversion, and the level of alkyne-Cer is significantly lower (Fig. S1). The rate of alkyne-PC synthesis was also reduced, although this reduction was not significant owing to the high interexperiment variability of the alkyne-PC signal for the parental HaCaT cells. Taken together, these results indicated that lipid metabolism is altered in HaCaT cells constitutively expressing pUL21. However, it remained unclear whether these differences reflected adaptations of cellular lipid metabolism in response to constitutive pUL21 expression rather than a direct effect upon CERT activity. Since CERT-mediated nonvesicular transport of Cer from the ER to the TGN defines the rate of Cer-to-SM conversion (21, 22, 23), pUL21-directed CERT dephosphorylation (and thus activation) should increase the rate of SM synthesis. Consistent with this hypothesis, the abundance of alkyne-SM as a fraction of total alkyne-Cer plus alkyne-SM was significantly higher in HaCaT21 *versus* HaCaT cells at early time points (0–30 min) during the chase (Fig. 1*D*). By the 60 min chase time point, the rate of alkyne-SM accumulation slows and the difference in relative abundance between cell lines was diminished, consistent with the alkyne-SM levels in both cell types approaching equilibrium. This increase in alkyne-SM accumulation as a fraction of alkyne-Cer plus alkyne-SM abundance was observed despite similar overall signal for alkyne-SM and alkyne-Cer in HaCaT21 cells when compared with parental cells at early time points (0–30 min), the abundance of these lipids being significantly lower in HaCaT21 cells at later time points (Fig. 1*E*). Reduced overall levels of SM plus Cer in HaCaT21 cells likely result from adaptation to constitutive pUL21 expression (and thus constitutive CERT hyperactivation) *via* stimulation of feedback pathways that reduce Sph to Cer conversion and/or increased back conversion of SM and/or Cer to Sph. Treatment of HaCaT cells with the highly specific CERT inhibitor HPA-12 (44) confirmed that the rate of Cer-to-SM conversion was defined by CERT activity: SM synthesis was significantly decreased in HPA-12–treated cells when compared with untreated cells, and there was concomitant accumulation of alkyne-Cer in treated cells (Figs. 1, *B*–*D* and S1). Immunoblot analysis of protein samples that were resolubilized following their precipitation during lipid extraction (Fig. 1*F*) confirmed that pUL21 was expressed and that dephosphorylated CERT (CERT^O^) predominates in HaCaT21 cells, in contrast to the parental and HPA-12–treated HaCaT cells.

**Figure 2:**
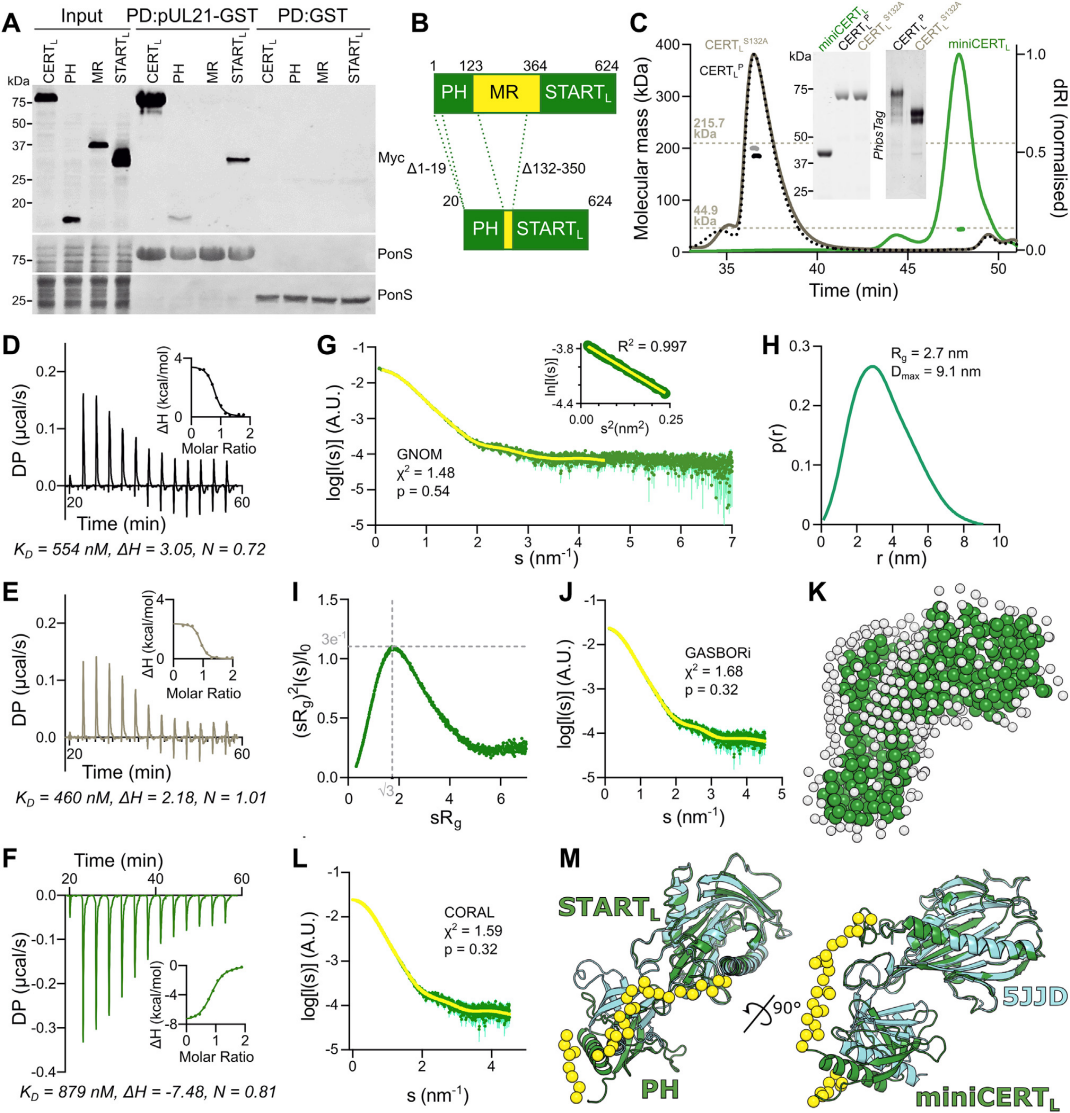
**pUL21 binds to the PH and START**_**L**_**domains of CERT**_**L**_**and to a monomeric form of CERT**_**L**_**comprising just the PH and START**_**L**_**domains (miniCERT**_**L**_**).***A*, minimal binding elements of CERT_L_ were determined *via* pull down (PD) experiment using immobilized purified pUL21-GST or GST alone to capture myc-tagged full-length CERT_L_ or truncations thereof expressed *via in vitro* transcription/translation. Captured proteins were subjected to SDS-PAGE and immunoblotting using an anti-myc antibody. Ponceau S (PonS) staining of the immunoblot membrane before blocking shows equal and efficient capture of the bait proteins across the tested conditions. *B*, schematic representation of miniCERT_L_. *Dotted lines* indicate the regions of full-length CERT_L_ that were omitted. *C*, SEC–MALS elution profiles (normalized differential refractive index, dRI) of StrepII-CERT_L_ (*black dotted*), StrepII-CERT_L_^S132A^ (*gray solid*), and H_6_-miniCERT_L_ (*green solid*). Weight-averaged molecular masses (*colored solid lines*) are shown across the elution peaks. The expected molecular masses for trimeric StrepII-CERT_L_ and StrepII-CERT_L_^S132A^, and for monomeric H_6_-miniCERT_L_, are shown as *dotted horizontal lines*. *Inset* shows Coomassie-stained SDS-PAGE of the samples used for SEC–MALS (*left*), plus SDS-PAGE supplemented with PhosTag reagent (*right*) of the StrepII-CERT_L_ proteins. *D*–*F*, representative ITC titration curve of pUL21-H_6_ binding to (*D*) StrepII-CERT_L_, (*E*) StrepII-CERT_L_^S132A^, and (*F*) H_6_-miniCERT_L_. *Insets* show normalized binding curves with integrated changes in enthalpy (ΔH) as a function of molar ratio. The affinity (*K*_*D*_), ΔH, and stoichiometry (N) for the presented titrations are displayed below. *G*, SAXS profile measured for H_6_-miniCERT_L_. The reciprocal-space fit of the *p*(*r*) profile to the SAXS data is shown as a *yellow line*. χ2, fit quality; p, Correlation Map (CorMap) probability of systematic deviations between the model fit and the scattering data (92). *Inset* shows the Guinier plot (*sR*_g_ < 1.3), which is linear as expected for an aggregate- and repulsion-free system. *H*, the real-space distance distribution function, *p*(*r*), calculated from the SAXS profile. *I*, dimensionless Kratky plot of the SAXS data. *Gray dotted lines* indicate the expected maximum of the plot for a compact protein (*sR*_g_ = √3, (*sR*_g_)^2^*I*(*s*)/*I*(0) = 3e^−1^) (114). *J*, fit of an *ab initio* dummy-residue model calculated using GASBOR to the SAXS profile and (*K*) representative GASBOR dummy-residue model. *White spheres* indicate modeled water beads of the hydration shell. *L*, fit to the SAXS profile of the pseudoatomic model of H_6_-miniCERT_L_ obtained using CORAL. *M*, CORAL pseudoatomic model of H_6_-miniCERT_L_ (*green ribbons*) superimposed onto the crystal structure of the complex between the CERT PH and START domains (*cyan ribbons*, PDB ID: 5JJD) (46) by aligning the START/START_L_ domains. Superposition is shown in orthogonal orientations where the linker regions or termini that were modeled by CORAL are depicted as *yellow spheres*. DP, differential power; GST, glutathione-*S*-transferase; ITC, isothermal titration calorimetry; PDB, Protein Data Bank; PH, Pleckstrin homology; SAXS, small-angle X-ray scattering; SEC–MALS, size-exclusion chromatography with inline multiangle light scattering; START, steroidogenic acute regulatory–related lipid transfer.

pUL21 has multiple functions during infection, promoting the PP1-mediated dephosphorylation of not just CERT but of multiple different cellular and viral proteins (18). It was therefore essential to identify a point mutant of pUL21 with impaired binding to CERT, but not to other cellular or viral partners, to dissect the functional significance of increased CERT activity during infection. Defining the interaction surfaces of transient protein complexes presents a considerable challenge (45), made more difficult in the case of pUL21 and CERT by their containing multiple domains and regions of intrinsic disorder (18). The minimal region of CERT required for pUL21 binding was thus probed using pull-down experiments where pUL21-glutathione-*S*-transferase (GST) purified following bacterial expression (bait) was used to capture CERT or truncations thereof (preys) expressed by *in vitro* coupled transcription/translation in wheat germ extract (Fig. 2*A*). The longer cytoplasmic isoform of CERT (CERT_L_), where expression of exon 11 extends the amino-terminal region of the START domain by 26 amino acids (START_L_), was used since both CERT and CERT_L_ have been shown to possess Cer transfer activity (23), and a contribution of these additional 26 amino acids to pUL21 binding could not be excluded. Full-length CERT_L_ was efficiently captured by pUL21-GST. The PH and START_L_ domains were also captured by pUL21-GST when expressed individually, albeit less efficiently, suggesting that the CERT_L_ possesses multiple pUL21-binding motifs. The CERT MR was not captured by pUL21-GST and is thus likely to be dispensable for binding. A truncated CERT_L_ construct that retained the pUL21-binding PH and START_L_ domains, but lacked the majority of the highly flexible MR, was thus designed for use in subsequent biochemical and structural studies (miniCERT_L_) (Fig. 2*B*). The extent of the MR retained to link the two domains was informed by the crystal structure of the CERT PH–START domain complex (46), defining a minimal distance of at least 55 Å between the PH domain C terminus and the START domain N terminus, and the desire to exclude the predicted coiled-coil region that may cause oligomerization (26). MR residues 132 to 350 were thus excluded from miniCERT_L_. The first 19 amino acids of the PH domain were also omitted from miniCERT_L_ as this region is predicted to be highly disordered and was excluded in previous structural studies (24, 47).

Previous studies identified that the MR mediates CERT trimerization (26, 48). Size-exclusion chromatography with inline multiangle light scattering (SEC–MALS) (Fig. 2*C*) confirmed that hyperphosphorylated StrepII-tagged CERT_L_^P^, purified following expression in mammalian (Freestyle 293F) cells, has an observed mass (184.3 kDa) approaching that expected for a trimer (215.7 kDa). Similarly, a constitutively hypophosphorylated form of CERT_L_ where S132 is mutated to alanine, and thus cannot become phosphorylated to initiate the serine-rich motif phosphorylation cascade (28), had an observed mass (199.2 kDa) approaching that expected for a trimer. In contrast, H_6_-miniCERT_L_ purified following bacterial expression was predominantly monomeric and monodisperse (observed mass of 43.9 kDa, expected monomeric mass of 44.9 kDa), consistent with trimerization of CERT_L_ being driven by the MR and not being dependent upon CERT_L_ phosphorylation.

Isothermal titration calorimetry (ITC) demonstrated that pUL21-H_6_ (18) binds StrepII-CERT_L_ with approximately micromolar affinity (Fig. 2, *D* and *E*, Tables 1 and S1), the observed affinity not differing significantly between the hyperphosphorylated (CERT_L_^P^) and hypophosphorylated (CERT_L_^S132A^) forms of the protein. While StrepII-CERT_L_^S132A^ forms a 1:1 complex with pUL21, the observed binding stoichiometry (N) was consistently lower for StrepII-CERT_L_^P^ (0.74), consistent with a proportion of the pUL21 binding sites on CERT_L_ being sterically occluded when the protein is hyperphosphorylated. H_6_-miniCERT_L_ and pUL21-H_6_ form an equimolar complex, binding with micromolar affinity similar to the full-length protein (Fig. 2*F* and Table 1). However, the thermodynamics of binding differ significantly; whereas pUL21 binding to CERT_L_ is endothermic and entropically driven, binding to miniCERT_L_ is exothermic and enthalpically driven with minimal change in overall entropy (Table 1). This is consistent with CERT_L_ undergoing significant conformational rearrangement upon binding to pUL21, whereas the conformational changes to miniCERT_L_ (if any) upon pUL21 binding are likely to be much more modest.

**Table 1:** Thermodynamic properties of the pUL21–CERT_L_ interaction

Syringe	Cell	*K*_*D*_ (nM)	ΔH (kcal/mol)	ΔG (kcal/mol)	−TΔS (kcal/mol)	N (stoichiometry)	n (replicates)
CERT_L_^P^	pUL21	772.7 ± 274.0	3.89 ± 1.29	−8.37 ± 0.20	−12.27 ± 1.07	0.74 ± 0.02	3
CERT_L_^S132A^	pUL21	642.0 ± 257.4	2.385 ± 0.29	−8.475 ± 0.25	−10.85 ± 0.06	0.97 ± 0.04	2
miniCERT_L_	pUL21	1042.0 ± 537.7	−8.65 ± 0.63	−8.21 ± 0.24	0.44 ± 0.78	0.89 ± 0.06	6
miniCERT_L_	pUL21C	4325.0 ± 1661.7	−3.84 ± 0.02	−7.35 ± 0.23	−3.51 ± 0.25	0.97 ± 0.04	2
miniCERT_L_	pUL21^V382E^	8086.7 ± 932.8	−7.20 ± 0.05	−6.95 ± 0.06	0.26 ± 0.02	1.16 ± 0.13	3

As quantitated by ITC. Experiments were performed n (replicates) times and mean ± SD values are shown. Data for individual titrations are presented as supporting information (Table S1).

Small-angle X-ray scattering (SAXS) in batch mode was used to probe the solution structure of H_6_-miniCERT_L_ (Fig. 2*G* and Table S2). The frequency distribution of real-space distances (*p*(*r*) profile) is largely symmetric (Fig. 2*H*), and the dimensionless Kratky plot (Fig. 2*I*) has a bell-shaped peak at *sR*_*g*_ = √3, consistent with miniCERT_L_ having a compact globular conformation in solution. *Ab initio* modeling of the H_6_-miniCERT_L_ scattering profile using GASBOR (49) yields a dummy-residue model (Fig. 2, *J* and *K*) that closely resembles the crystal structure of CERT START and PH domain complex (46). A pseudoatomic model of the H_6_-miniCERT_L_ solution structure, generated using the crystal structures of the CERT PH and START domains determined in isolation (24, 25) as rigid bodies, confirms the similarity of the H_6_-miniCERT_L_ solution model to the crystal structure of the CERT PH–START domain complex (Fig. 2, *L* and *M*). Collectively, the MALS, ITC, and SAXS analyses demonstrate that miniCERT_L_ forms a compact monomeric protein with high affinity for pUL21, representing an excellent tool for structural studies.

**Figure 3:**
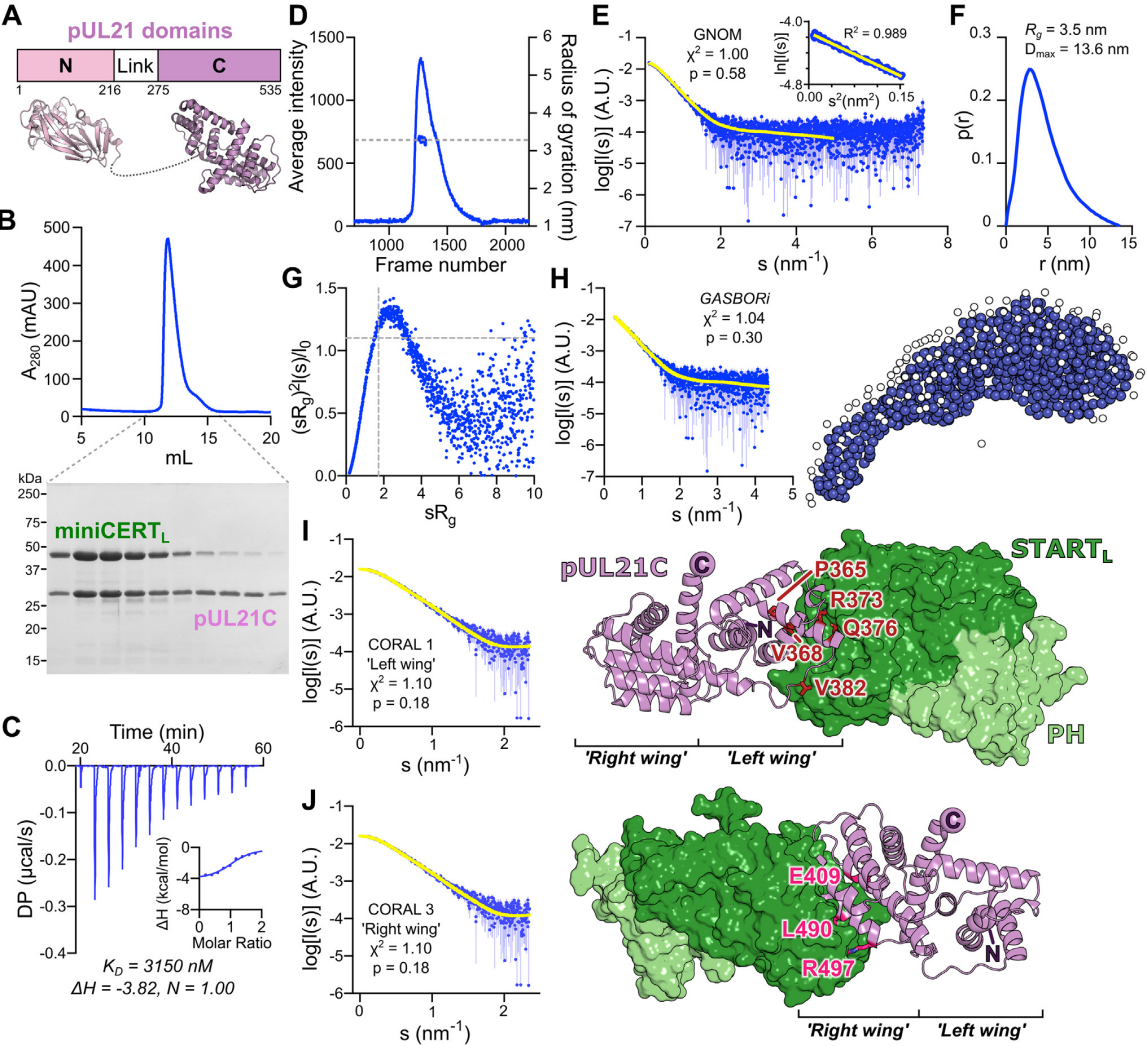
**Solution structure of the H**_**6**_**-pUL21C–H**_**6**_**-miniCERT**_**L**_**heterodimer.***A*, schematic of pUL21, with N- and C-terminal domains joined by a highly dynamic linker (18). Crystal structures of domains (N, PDB ID: 4U4H (50); C, PDB ID: 5ED7 (51)) are shown. *B*, Superdex 75 10/300 SEC elution profile of the H_6_-pUL21C–H_6_-miniCERT_L_ complex, preformed in the presence of 1.32-fold molar excess of H_6_-pUL21C. *Dotted lines* indicate fractions that were collected and subjected to SDS-PAGE analysis with Coomassie staining, revealing coelution of the two proteins. *C*, representative ITC titration curve of H_6_-pUL21C binding to H_6_-miniCERT_L_. *Inset* shows normalized binding curve with integrated changes in enthalpy (ΔH) as a function of molar ratio. The ranges of the *vertical axes* are identical to Figure 2*F*. The affinity (*K*_*D*_), ΔH, and stoichiometry (N) for the presented titration are displayed below. *D*, SEC elution profile (partial integrated scattered X-ray intensity *versus* data frame number) obtained during SEC–SAXS analysis of H_6_-pUL21C–H_6_-miniCERT_L_ complex. *Dashed line* indicates the calculated radius of gyration (*R*_g_) across the frames averaged for structural analyses. *E*, averaged SAXS profile of the H_6_-pUL21C–H_6_-miniCERT_L_ complex. The reciprocal-space fit of the *p*(*r*) profile to the SAXS data is shown as a *yellow line*. χ^2^, fit quality; *p*, Correlation Map (CorMap) probability of systematic deviations between the model fit and the scattering data (92). *Inset* displays the Guinier plot (*sR*_g_ < 1.3), which is linear as expected for an aggregate- and repulsion-free system. *F*, real-space distance distribution function, *p*(*r*), calculated from the SAXS profile. *G*, dimensionless Kratky plot of the SAXS data. *Gray dotted lines* indicate the expected maximum of the plot for a compact protein (*sR*_g_ = √3, (*sR*_g_)^2^*I*(*s*)/*I*(0) = 3e^−1^). *H*, *ab initio* modeling of H_6_-miniCERT_L_–H_6_-pUL21C using GASBOR. Fit of the calculated scattering (*yellow*) to the SAXS profile is shown, as is a representative dummy-residue model (*blue spheres*) with modeled water beads of the hydration shell (*white spheres*). *I* and *J*, pseudoatomic models of the H_6_-miniCERT_L_–H_6_-pUL21C complex generated using CORAL. The fits of the computed scattering (*yellow*) to the H_6_-pUL21C–H_6_-miniCERT_L_ SAXS profile (*blue*) are shown. High-quality fits are obtained with models where miniCERT_L_ binds either the *left* or *right* “wings” of the dragonfly-like pUL21C domain (51). pUL21C is shown as a *violet ribbon* with “wings” and termini labeled, and miniCERT_L_ as a *green* molecular surface (PH, *light green*; START_L_, *dark green*). For clarity, regions absent from the crystal structures that were modeled by CORAL are not displayed. Residues from the *left* and *right* “wings” of pUL21C that were selected for further investigation are shown as *sticks* with *red* (*I*) or *pink* (*J*) carbon atoms, respectively. An additional pseudoatomic model plus its fit to the H_6_-pUL21C–H_6_-miniCERT_L_ SAXS profile is shown in Figure S3, where the fits presented in (*I*) and (*J*) are also reproduced for ease of comparison. DP, differential power; ITC, isothermal titration calorimetry; PDB, Protein Data Bank; PH, Pleckstrin homology; SAXS, small-angle X-ray scattering; SEC, size-exclusion chromatography; START, steroidogenic acute regulatory–related lipid transfer.

Structural characterization *via* X-ray crystallography and SAXS has revealed pUL21 to comprise two domains joined by a highly flexible linker (Fig. 3*A*) (18, 50, 51), and immunoprecipitation experiments mapped CERT binding to the pUL21 C-terminal domain (18). The C-terminal domain of pUL21 (pUL21C), spanning amino acids 275 to 535, was expressed with an N-terminal hexahistidine tag and purified following bacterial expression. In contrast to a previous report (51), we did not observe copurification of nucleic acid with H_6_-pUL21C, the purified protein having an absorbance ratio of ∼0.6 at 260/280 nm. SEC–MALS and SAXS analysis (Fig. S2) confirmed that H_6_-pUL21C is monomeric and monodisperse, adopting a compact structure in solution that matches the previously determined crystal structure (χ^2^ = 0.99, CorMap *p* = 0.133) (51). SEC analysis of a preformed complex between purified H_6_-miniCERT_L_ and a 1.34-fold molar excess of H_6_-pUL21C confirmed that the two proteins form a stable complex (Fig. 3*B*), suitable for structural studies. ITC demonstrated that H_6_-pUL21C forms an equimolar complex with H_6_-miniCERT_L_ with a dissociation constant (*K*_*D*_) of 4.3 ± 1.2 μM (Fig. 3*C*, Tables 1 and S1). The approximately fourfold reduction in miniCERT_L_ binding by pUL21C *versus* full-length pUL21 is consistent with previous immunoprecipitation results, where transfected pUL21C-GFP captured endogenous CERT slightly less efficiently than did pUL21-GFP in human embryonic kidney 293T (HEK293T) cells (18). These ITC experiments confirm that the C-terminal domain of pUL21 is the major determinant of CERT binding. Attempts to predict the structure of pUL21C in complex with miniCERT_L_ using AlphaFold-Multimer (52) did not yield high confidence models, presumably because of a paucity of coevolutionary signal between these viral and human proteins, and structural characterization of this complex thus required an experimental approach.

To probe the structural basis of the CERT recruitment by pUL21, a preformed complex of H_6_-miniCERT_L_ and H_6_-pUL21C was subjected to SEC with inline SAXS measurement (SEC–SAXS, Fig. 3*D*). SAXS data were processed by averaging frames with a consistent calculated radius of gyration (*R*_g_) and then subtracting averaged buffer frames to yield the H_6_-miniCERT_L_–H_6_-pUL21C complex scattering profile (Fig. 3*E*). The probable frequency of real-space distances (*p*(*r*) profile) of the complex is moderately asymmetric (Fig. 3*F*), in contrast to the highly symmetric *p*(*r*) profiles of H_6_-pUL21C (Fig. S2*D*) or H_6_-miniCERT (Fig. 2*H*) alone, suggesting a less spherical particle, and the dimensions of the complex (*R*_g_ = 3.5 nm, *D*_max_ = 13.6 nm) are substantially larger than for H_6_-pUL21C (*R*_g_ = 2.2 nm, *D*_max_ = 8.5 nm) or H_6_-miniCERT (*R*_g_ = 2.7 nm, *D*_max_ = 9.1 nm). The peak of the dimensionless Kratky plot is slightly higher, with its peak away from *sR*_g_ = √3 (Fig. 3*G*), suggesting some flexibility in the system granted either by modest dissociation of the complex or some mobility of the H_6_-miniCERT_L_ domains with respect to each other and H_6_-pUL21C. *Ab initio* modeling using GASBOR indicated an elongated molecule (Fig. 3*H*). While initial pseudoatomic models of the complex generated using a fixed conformation of H_6_-miniCERT_L_ did not fit the SAXS profile acceptably, allowing the PH and START_L_ domains freedom to move with respect to each other and to H_6_-pUL21C yielded three pseudoatomic models with high-quality fits to the SAXS profile (Figs. 3, *I* and *J* and S3). In all three top models, miniCERT_L_ binds “end-on” to the pUL21 molecule, forming an ellipsoidal “rugby ball”–like particle. In two of the top three models, miniCERT_L_ binds the “*left wing*” of the dragonfly-shaped pUL21C domain (51), whereas in the other, it binds the “*right wing*” (Figs. 3, *I* and *J* and S3). All these models have a similar overall shape, and thus all explain the SAXS scattering data well, but the relative orientations of H_6_-miniCERT_L_ and H_6_-pUL21C differ. All three top models were thus used to design specific pUL21 mutations that might disrupt (mini)CERT binding.

**Figure 4:**
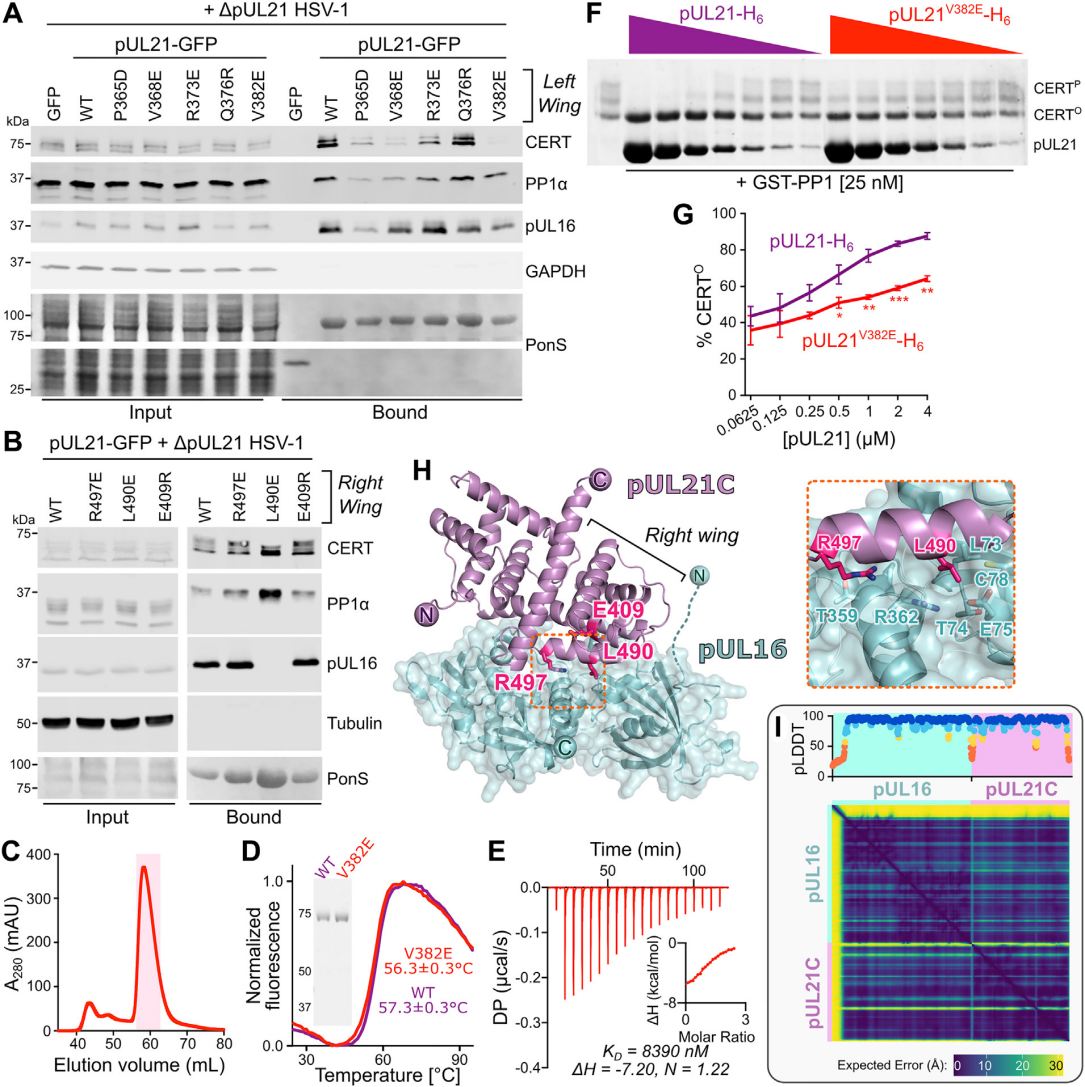
**Identification of pUL21 point mutants with disrupted binding to CERT or pUL16.***A* and *B*, immunoblot following immunoprecipitation from HEK293T cells transfected with plasmids encoding GFP-tagged pUL21, either WT or with amino acid substitutions at the putative CERT_L_ binding interface on the *left* (*A*) or *right* (*B*) wings of pUL21C, or encoding GFP alone. At 24 h post-transfection, cells were infected with ΔpUL21 HSV-1 (MOI = 5), and at 16 h postinfection, cells were lysed, tagged proteins were captured using GFP affinity resin, and the bound proteins were subjected to SDS-PAGE and immunoblotting using the antibodies listed. Ponceau S (PonS) staining of the nitrocellulose membrane before blocking is shown to confirm efficient capture of GFP-tagged proteins. GAPDH (*A*) and tubulin (*B*) are used as loading controls. *C*, Superdex 75 16/600 SEC elution profile of pUL21^V382E^-H_6_ following bacterial expression and affinity capture. Indicated peak was pooled and used for subsequent experiments. *D*, differential scanning fluorimetry of WT (*purple*) and V382E (*red*) pUL21-H_6_. Representative curves are shown, and melting temperature (*T*_m_) is mean ± SD of three technical replicates. *Inset* shows Coomassie-stained SDS-PAGE of the purified proteins. *E*, representative ITC titration curve of pUL21^V382E^-H_6_ binding to H_6_-miniCERT_L_. *Inset* shows normalized binding curve with integrated changes in enthalpy (ΔH) as a function of molar ratio. The ranges of the *vertical axes* are identical to Figure 2*F*. The affinity (*K*_*D*_), ΔH, and stoichiometry (N) for the presented titration are displayed below. *F*, *in vitro* dephosphorylation assay using all-purified proteins. About 0.5 μM CERT^P^ was incubated with varying concentrations of pUL21-H_6_ WT or V382E (twofold dilution from 4 μM to 62.5 nM) in the presence of 25 nM GST-PP1 for 30 min at 30 °C. Proteins were resolved using SDS-PAGE supplemented with PhosTag reagent to enhance separation of CERT that is hyperphosphorylated (CERT^P^) or hypophosphorylated (CERT^O^), and protein bands were visualized using Coomassie. Images are representative of two independent experiments. *G*, quantitation of concentration-dependent pUL21-mediated stimulation of CERT dephosphorylation as determined by densitometry. Ratio of CERT^O^ to total CERT (CERT^O^ + CERT^P^) for two independent experiments is shown (mean ± SD). Data points are labeled if significantly different: ∗*p* < 0.05; ∗∗*p* < 0.01; and ∗∗∗*p* < 0.001 (two-way ANOVA with Sidak’s multiple comparisons test). *H*, predicted structure of pUL21C (*violet ribbon*) in complex with pUL16 (*cyan ribbon and surface*). Residues 1 to 34 of pUL16, which are predicted to be disordered, are not shown. Residues of the pUL21 *right* wing that were substituted in (*B*) are shown as *sticks*. *Inset* shows selected pUL16 amino acids residues in close proximity to L490 and R497 of pUL21. *I*, per-residue predicted Local Distance Difference Test (pLDDT) scores (*top*) and predicted aligned error (PAE) matrix (*bottom*) for predicted pUL16–pUL21C complex. DP, differential power; HEK293T, human embryonic kidney 293T cell line; HSV-1, herpes simplex virus 1; ITC, isothermal titration calorimetry; MOI, multiplicity of infection; PP1, protein phosphatase 1; SEC, size-exclusion chromatography.

Mutations in pUL21C were designed to identify whether miniCERT_L_ binds the *left wing* or *right wing* of this domain. Amino acids in helix α4 or the subsequent loop of pUL21C were mutated to test binding to the *left wing* (Fig. 3*I*), whereas amino acids in helices α5 and α9 were used to test binding to the *right wing* (Fig. 3*J*). Immunoprecipitation experiments in transfected HEK293T cells that had been infected with HSV-1 lacking pUL21 expression (HSV-1 ΔpUL21) demonstrated that four of five substitution in the *left wing* of pUL21C (P365D, V368E, R373E, and V382E) disrupted the ability of CERT to coprecipitate with pUL21-GFP (Fig. 4*A*), whereas none in the pUL21C *right wing* disrupted CERT coprecipitation (Fig. 4*B*). These results are consistent with CERT binding the *left wing* of pUL21C. They are also consistent with observations that CERT binding is lost when pUL21C has a bulky N-terminal GFP tag but retained when the GFP tag is C terminal (18), as the *left-wing* models place the pUL21C amino terminus but not the carboxy terminus in close proximity to miniCERT_L_ (Figs. 3*I* and S3).

Of the pUL21 substitutions that disrupted CERT binding (Fig. 4*A*), V382E appeared to cause the largest decrease in CERT binding while maintaining the ability of pUL21 to coprecipitate its other known binding partners, PP1 (18) and pUL16 (10). The presence of a valine residue at this position is absolutely conserved in HSV-1 and HSV-2 and is highly conserved across members of the simplexvirus genus (Fig. S4). H_6_-pUL21^V382E^ was purified following bacterial expression (Fig. 4*C*). Differential scanning fluorimetry (a.k.a. Thermofluor) showed pUL21^V382E^ to be well folded as its thermal stability is similar to WT H_6_-pUL21 (Fig. 4*D*). ITC analysis demonstrated that H_6_-pUL21^V382E^ has approximately eightfold reduced binding affinity for H_6_-miniCERT_L_ when compared with WT pUL21 (Fig. 4*E*, Tables 1 and S1). The effect of the V382E substitution upon the ability of pUL21 to promote CERT dephosphorylation, converting CERT^P^ to CERT^O^, was probed using an *in vitro* dephosphorylation assay with all-purified reagents (Fig. 4, *F* and *G*). The dose-dependent acceleration of GST-PP1–mediated CERT dephosphorylation is significantly greater for WT pUL21 than pUL21^V382E^ (*p* = 0.040; two-way ANOVA with Sidak’s multiple comparison test), consistent with the GST-PP1–pUL21^V382E^ complex having lower affinity for the substrate CERT^P^ (EC_50_[pUL21] = 0.491 ± 0.123 μM, EC_50_[pUL21^V382E^] = 3.329 ± 0.7874 μM; three parameter dose–response curve, n = 2 independent experiments).

**Figure 5:**
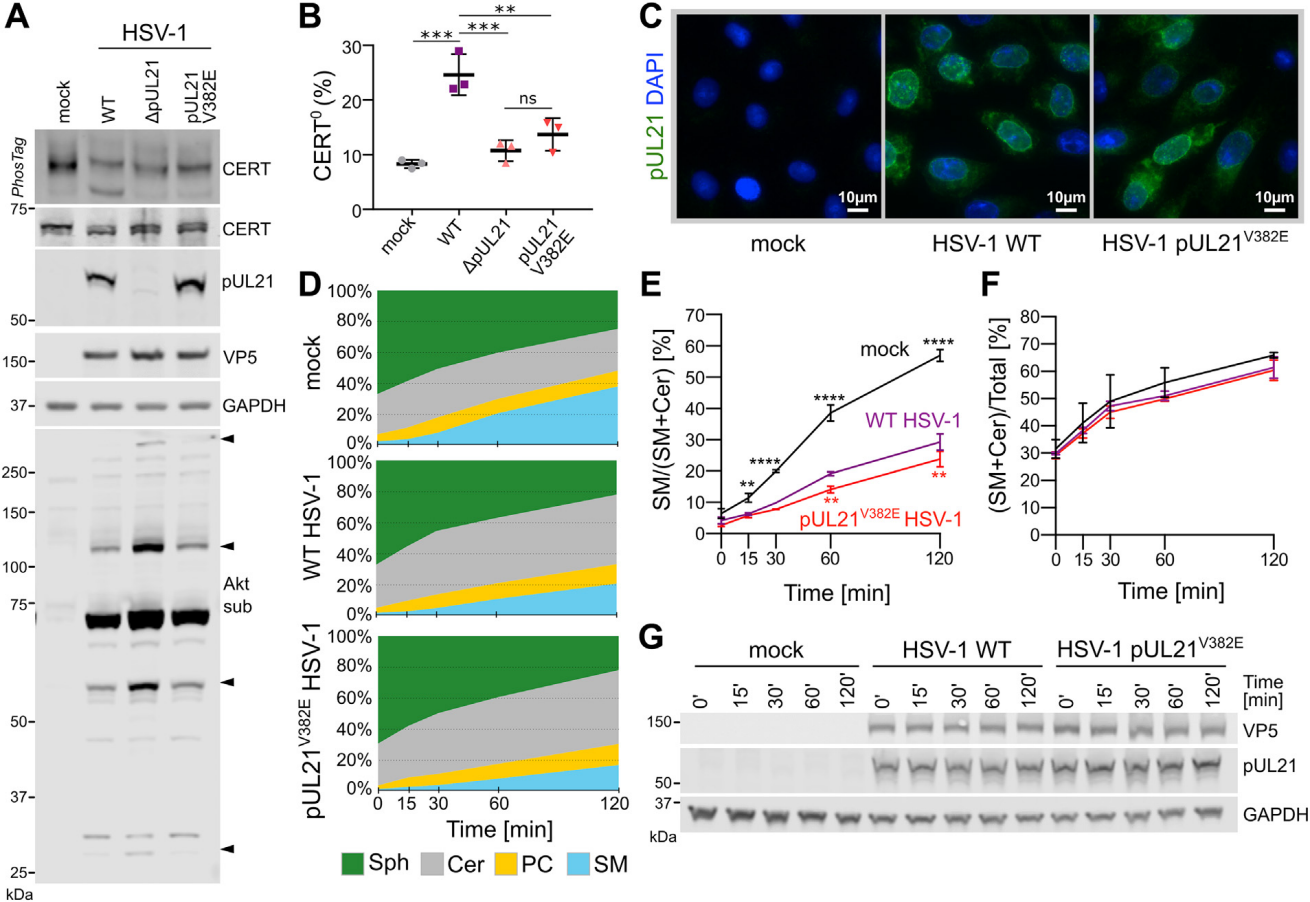
**Mutating the CERT-binding interface of pUL21 inhibits CERT dephosphorylation and reduces the rate of sphingomyelin synthesis in infected cells.***A*, HaCaT cells were infected at MOI = 5 with WT HSV-1, HSV-1 lacking pUL21 expression (ΔpUL21), or a pUL21 point mutant virus (pUL21^V382E^). Lysates were harvested at 16 hpi in the presence of phosphatase inhibitors and subjected to SDS-PAGE plus immunoblotting using the antibodies listed. Where indicated, the gel was supplemented with PhosTag reagent to enhance separation of CERT phosphoforms. The antibody recognizing phosphorylated Akt substrates (Akt sub) illustrates activity of the HSV-1 kinase pUS3, several substrates of which are dephosphorylated in a pUL21-dependent manner (*arrowheads*) (18). VP5, infection control. *B*, quantitation of the CERT dephosphorylation level (ratio of CERT^O^ to total CERT) in cells infected with WT or mutant HSV-1, as determined by densitometry. Results are presented as mean ± SD from three independent experiments. One-way ANOVA with Tukey’s multiple comparisons test was used for the statistical analysis (ns, nonsignificant; ∗∗*p* < 0.01; ∗∗∗*p* < 0.001). *C*, Vero cells were infected at MOI = 1, fixed at 14 hpi, and stained with DAPI (*blue*) plus an antibody recognizing pUL21 (*green*). *D*, pulse-chase experiment to measure the rate of Sph conversion to Cer, SM, and PC. HaCaT cells were infected with WT or pUL21^V382E^ HSV-1 at MOI = 5 or mock infected. Cells were incubated with 5 μM alkyne-Sph (pulse) at 14 hpi for 5 min and harvested for lipid extraction either immediately (0 min) or at the indicated times (chase). Extracted lipids were bioconjugated to 3-azido-7-hydroxycoumarin using click chemistry, separated by HPTLC, visualized using UV light, and relative lipid abundances were quantitated by densitometry. Data are from one representative experiment of two independent repeats. *E*, rate of SM synthesis expressed as its fraction in the cumulative signal for SM and Cer. *F*, the proportion of alkyne-Sph incorporated into either Cer or SM, representing the temporal influx of Sph into the SM biosynthesis pathway. *E* and *F*, the data represent two independent experiments (mean ± SD). Data points are labeled if significantly different to WT HSV-1: ∗∗*p* < 0.01; ∗∗∗∗*p* < 0.0001 (two-way ANOVA with Dunnett’s multiple comparisons test). *G*, the resolubilized proteins precipitated during lipid extraction were analyzed by SDS-PAGE and immunoblotting using the antibodies listed. Cer, ceramide; DAPI, 4′,6-diamidino-2-phenylindole; hpi, hours postinfection; HPTLC, high-performance TLC; HSV-1, herpes simplex virus 1; MOI, multiplicity of infection; PC, phosphatidylcholine; SM, sphingomyelin; Sph, sphingosine.

While mutation of the pUL21C *right wing* did not prevent CERT binding, one substitution (L490E) abolished binding to pUL16 (Fig. 4*B*). pUL21^L490E^ retains the ability to bind CERT and PP1, suggesting that the mutant is stable and maintains a correct overall fold. A model of pUL16 in complex with pUL21C was generated using AlphaFold-Multimer (52), revealing that pUL16 is predicted to bind the *right wing* of pUL21C, with helix α9 spanning residues 486 to 497 being central to this interaction (Fig. 4*H*). The structures of pUL16 and pUL21C, and their relative orientations in the complex, were predicted with high overall confidence (Fig. 4*I*). pUL16 is predicted to comprise two domains, an N-terminal domain (residues 35–172) that is joined by a short linker to a C-terminal domain (residues 179–371) (Fig. S5*A*). A structural homology search using DALI (53) revealed that the pUL16 C-terminal domain shares greatest structural similarity to the N-terminal domain of pUL21 (Fig. 5*B* and Table S3), whereas the pUL16 C-terminal domain is similar in fold to a variety of bacterial and eukaryotic proteins that have diverse annotated functions ranging from phosphatase activity to lipid binding (Fig. 5, *C* and *D* and Table S3). Residues 1 to 34 of pUL16 were predicted with low confidence and are likely to be intrinsically disordered. Residue L490 of pUL21C is predicted to bind a hydrophobic pocket formed by L91, R362, and residues 73 to 78 of pUL16 (Fig. 4*H*). Loss of pUL16 binding by pUL21C L490E is consistent with the introduction of a charged glutamic acid side chain into this pocket being energetically unfavorable. pUL21 R497 is in close proximity to pUL16 residues T359 and R362. Inverting the charge of pUL21 residue 497 by substituting arginine for glutamate did not prevent pUL16 binding, presumably because the negative glutamic acid side chain could still form polar interactions with these residues. pUL21 E409 is not predicted to lie at the pUL16 binding interface, consistent with mutation of this residue not affecting the pUL21C–pUL16 interaction.

To probe the ability of the pUL21 mutant with reduced CERT binding (pUL21^V382E^) to stimulate CERT dephosphorylation during infection, a mutant strain of HSV-1 encoding pUL21^V382E^ was generated using two-step Red recombination (54). Dephosphorylated CERT (CERT^O^) is significantly less abundant in HaCaT cells infected with HSV-1 expressing pUL21^V382E^ or lacking pUL21 expression (ΔpUL21) when compared with WT HSV-1-infected cells (Fig. 5, *A* and *B*). In addition to CERT, pUL21 expression reduces the phosphorylation of multiple substrates of the viral kinase pUS3 in HSV-1–infected cells (18), the phosphorylated forms of these substrates being detectable using an antibody that recognizes phosphorylated substrates of the cellular kinase Akt (55). While infection with ΔpUL21 HSV-1 causes a dramatic increase in the abundance of multiple phosphorylated pUS3 substrates, the abundance of these phosphoforms is indistinguishable between WT and pUL21^V382E^ HSV-1 (Fig. 5*A*). This confirms that the V382E substitution specifically disrupts CERT dephosphorylation, rather than generally inhibiting the ability of pUL21^V382E^ to recruit PP1 to substrates. Similar changes in CERT dephosphorylation, but not in the dephosphorylation of other pUS3 substrates, are observed when Vero cells are infected with pUL21^V382E^ HSV-1 (Fig. S6, *A* and *B*). Immunocytochemistry confirms that both WT and V382E pUL21 have the similar subcellular localization, being observed predominantly at the nuclear rim of infected Vero cells (Fig. 5*C*).

Metabolic labeling was used to monitor the impact of pUL21-mediated CERT dephosphorylation on sphingolipid biogenesis during infection. A pulse-chase experiment was performed where HaCaT cells infected with WT or pUL21^V382E^ HSV-1, or mock infected, were incubated for 5 min with alkyne-Sph at 14 h postinfection (hpi), and its metabolites were monitored for 2 h. Cer accumulates, and the rate of SM synthesis is significantly decreased in cells infected with WT and pUL21^V382E^ HSV-1 when compared with uninfected cells (Fig. 5*D*). Although a substantial decrease in the rate of SM synthesis is seen for both WT and mutant HSV-1 infection, the defect is significantly larger in cells infected with HSV-1 pUL21^V382E^ (Fig. 5*E*). The overall abundance of SM plus Cer is similar between infected and uninfected cells, suggesting that Cer to SM conversion is specifically impaired rather than influx of alkyne-Sph into the SM biogenesis pathway being defective (Fig. 5*F*). Taken together, this suggests that pUL21-mediated activation of CERT accelerates Cer to SM conversion during infection, albeit from a much lower base than in uninfected cells.

**Figure 6:**
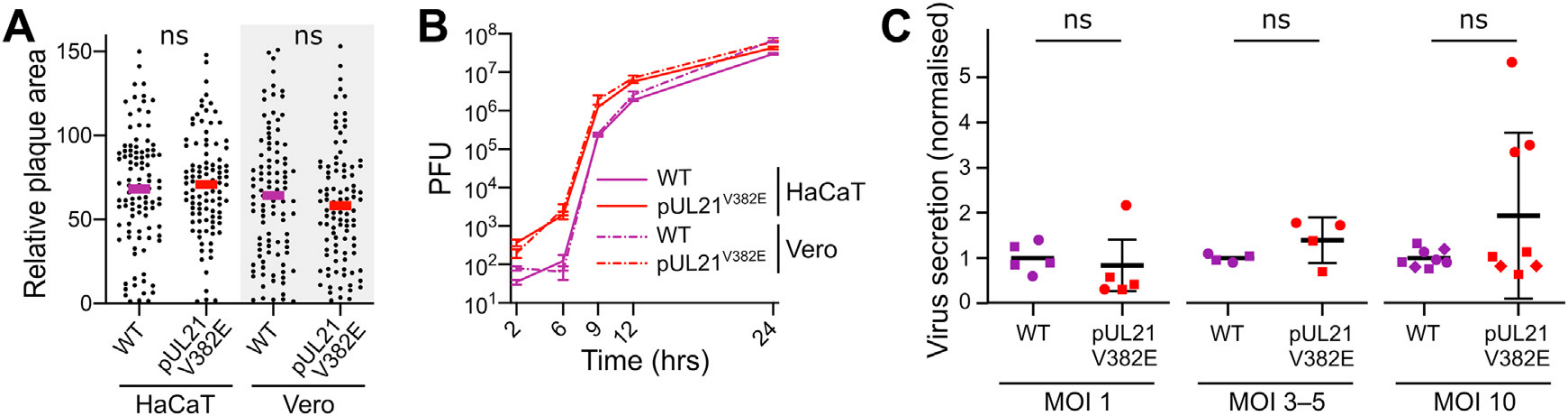
**pUL21-mediated dephosphorylation of CERT does not enhance virus replication, secretion, or spread in cultured cells.***A*, monolayers of HaCaT or Vero cells were infected with 100 plaque-forming units of WT or pUL21^V382E^ HSV-1. Following infection, cells were overlaid with medium containing 0.6% carboxymethyl cellulose and incubated for 48 h, and then fixed and immunostained with chromogenic detection. Relative plaque areas (pixels) were measured using Fiji (102, 103). *Bars* represent mean plaque sizes, which were compared using unpaired *t* test (n = 100; ns). *B*, single-step (high MOI) growth curves of WT and pUL21^V382E^ HSV-1. Monolayers of HaCaT (*continuous line*) or Vero (*dotted line*) cells were infected (MOI = 5) with the viruses shown. Samples were harvested at the indicated times, and titers were determined by plaque assay using Vero cells. Data are presented as mean values ± SD of technical duplicates from one representative experiment. Difference in replication kinetics across two biological replicates is not statistically significant (two-way ANOVA with Sidak’s multiple comparisons test). *C*, virus release into the culture supernatant from HaCaT cells infected with WT or pUL21^V382E^ HSV-1 at various MOIs. Samples were harvested at 12 hpi and virus infectivity in the cells *versus* the culture medium was measured by titration on Vero cells. The fold change in secretion of infectivity into the culture medium for pUL21^V382E^*versus* WT HSV-1 is shown as mean values ± SD of two (MOI = 1 or 3–5) or three (MOI = 10) independent experiments. Technical replicates for each independent experiment are grouped by shape. For MOI 3 to 5, the data represent one independent experiment performed at MOI = 3 (*squares*) and one at MOI = 5 (*circles*). For each MOI, the extent of virus secretion was compared using an unpaired two-tailed *t* test with Welch’s correction (ns). HSV-1, herpes simplex virus 1; MOI, multiplicity of infection; ns, nonsignificant.

Having confirmed that pUL21^V382E^ HSV-1 specifically lacks the ability to stimulate CERT dephosphorylation, and that HSV-1 encoding pUL21^V382E^ has a reduced rate of Cer to SM conversion, the impact of this deficit upon virus replication and spread in cultured cells was assessed. WT and pUL21^V382E^ HSV-1 form similar-sized plaques on HaCaT and Vero cells (Fig. 6*A*), suggesting that CERT dephosphorylation is dispensable for efficient viral cell-to-cell spread. A single-step growth curve, where Vero and HaCaT cells are infected at a high multiplicity of infection (MOI) and the production of infectious progeny is monitored over time, was used to compare the replication of WT and pUL21^V382E^ HSV-1 (Fig. 6*B*). Both viruses produce similar abundance of infectious progeny by 24 h postinfection. In two biologically independent experiments performed for each cell type, the kinetics of virus replication appeared to be accelerated, with higher titers of pUL21^V382E^ HSV-1 being observed between 6 and 12 h postinfection, but the difference in growth rate was not statistically significant for either cell line (two-way ANOVA with Sidak’s multiple comparisons test).

While HSV-1 preferentially remains cell attached, spreading *via* direct cell–cell contacts, the cell-free secretion of virions from infected cells is altered when CERT is depleted or overexpressed (38). Cell-free release of pUL21^V382E^ HSV-1 was determined by quantitating the amount of infectivity secreted into the medium as a percentage of overall infectivity (secreted plus cell associated), normalized for each independent replicate to the secretion of a WT virus in control experiments performed at the same time. HaCaT cells infected for 12 h with various MOI of pUL21^V382E^ HSV-1 did not release significantly more or less infectivity when compared with WT HSV-1 infection (Fig. 6*C*). Similarly, the cell-free release of infectivity at 12 hpi did not differ between Vero cells infected with pUL21^V382E^ or WT HSV-1 (Fig. S6*C*).

## Discussion

HSV-1 extensively remodels the proteome of infected cells, altering both the abundance (3) and post-translational modification status (2, 18, 56) of multiple cellular proteins. We know much less about HSV-1-mediated changes to the cellular lipidome, although previous studies have identified that HSV-1 infection changes phosphoinositide levels (57) and increases the rate of *de novo* phospholipid synthesis (58). By combining biochemical and structural studies with cell-based models of infection, we show here that pUL21 accelerates the conversion of Cer to SM in infected cells by promoting dephosphorylation and activation of the Cer transport protein CERT (Fig. 5). This is the first observation of a viral protein directly binding CERT and altering its activity. While pUL21-mediated CERT activation causes an apparent modest acceleration in the rate of virus replication, this effect is not statistically significant and CERT activation does not contribute to replication or cell-to-cell spread of HSV-1 in cultured keratinocytes or epithelial cells (Fig. 6).

Our previous work showed that pUL21 is a phosphatase adaptor with multiple different targets (18). Mutation of the pUL21 TROPPO motif, required for PP1 binding and thus stimulation of dephosphorylation, dramatically reduced the replication and spread of HSV-1. While the TROPPO motif was identified *via* its conservation across alphaherpesvirus sequences, the molecular basis of CERT recruitment remained unknown. Our previous immunoprecipitation experiments demonstrated that the N-terminal domain of pUL21 was dispensable for CERT binding (18). Biochemical mapping and SAXS structural characterization presented here have now identified a specific mutation, V382E, that decreases the affinity of pUL21 for miniCERT_L_ by approximately eightfold (Fig. 4*E*). While this reduction in affinity is moderate when assayed in dilute biochemical solution, it is sufficient to disrupt coprecipitation of CERT from transfected cells (Fig. 4*A*). The V382E substitution abolishes pUL21-mediated dephosphorylation of CERT during infection (Fig. 5, *A* and *B*) and significantly decreases the rate of Cer to SM conversion (Fig. 5*E*). These results are consistent with other binding partners competing to bind CERT and/or pUL21 within the context of infected cells, these competing interactions amplifying the effect of reduced pUL21–CERT binding affinities. The V382E mutation does not prevent pUL21 binding to pUL16 (Fig. 4*A*) nor the ability of pUL21 to stimulate dephosphorylation of other targets (Fig. 5*A*), confirming that pUL21 binds CERT *via* a molecular surface that is distinct from the binding site(s) of other targets. The WT levels of virus replication and cell-to-cell spread observed for pUL21^V382E^ HSV-1 are consistent with our previous *in vitro* evolution studies of HSV-1 mutants where the pUL21 TROPPO motif was mutated (18). Adaptation of the virus to the loss of pUL21 PP1 binding, *via* suppressor mutations that reduce the activity of the kinase pUS3, restored virus replication and spread without restoring enhanced CERT dephosphorylation (18). We therefore conclude CERT is not a critical substrate of pUL21 for virus replication and spread in cultured fibroblasts and keratinocytes. The C-terminal domain of pUL21 is highly conserved across HSV-1 and HSV-2 (>93% and 80–84% identity, respectively). V382 is absolutely conserved across these species, and a small hydrophobic residue (valine, leucine, or alanine) at this position is very highly prevalent across the genus *Simplexvirus* (Fig. S4*A*). The C-terminal domain of pUL21 is much less well conserved when considering the entire subfamily *Alphaherpesvirinae* (>20% sequence identity across the subfamily), with the amino acid residue at position 382 being correspondingly variable (Fig. S4*B*). This is consistent with our previous observation that the pUL21 homolog ORF38 from varicella-zoster virus (genus: *Varicellavirus*) does not bind CERT (18). It is therefore likely that CERT binding and activation is a conserved feature of HSV-1, HSV-2, and other simplexviruses but does not extend to other genera of alphaherpesvirus. Given the lack of phenotype observed for viruses encoding the CERT-binding pUL21^V382E^ mutant in cultured cells, it is possible that this interaction plays a role in immune regulation or transmission fitness (59). Alternatively, the interaction may enhance virus replication in other cell types. Specifically, we note that HSV-1 is a neurotropic virus and that sphingolipids like SM are highly enriched in neurons, where they are critical for correct neuronal development and function (31). Studying whether pUL21-mediated CERT dephosphorylation affects virus replication in neurons is an important question for future investigation.

Studies of cellular lipid metabolism are complicated by robust cellular feedback mechanisms (60) and challenges in accurately detecting changes in lipid abundance (61). Here, we successfully employed lipid labeling, click chemistry, and HPTLC to monitor the kinetics of Sph metabolism, revealing that HSV-1 pUL21 significantly increases the rate of CERT-mediated Cer to SM conversion both outside (Fig. 1) and within (Fig. 5) the context of infection. Furthermore, we observe a highly significant increase in the abundance of labeled Cer when cells are infected with HSV-1, this increase being greater when pUL21-mediated CERT activation has been abolished. The dramatic change in the rate of Cer to SM conversion is most likely explained by the known propensity of HSV-1 to promote dispersal of the Golgi and TGN (4, 62). Such dispersal would alter ER–TGN contact sites, where CERT-mediated transport enables Cer to SM conversion by TGN-resident SM synthase (63). Side-by-side kinetic comparison of lipid metabolic enzymes including Cer synthase confirms that the presence of alkyne groups within acyl tails does not significantly alter lipid catalysis when compared with radiolabeled or natural substrates (41). While the CERT START domain has been demonstrated to bind Cer containing an alkyne group plus a photoactivatable diazirine group (64, 65), we are unaware of any comparisons between CERT lipid transfer activity with natural or radiolabeled Cer *versus* alkyne-Cer. It is possible that the presence of an alkyne group may alter the affinity of CERT for Cer, and hence that pUL21-mediated CERT activation may cause a greater or lesser change to the rate of unlabeled Cer to SM conversion. Furthermore, our experimental strategy to probe CERT activity exploited the uptake of exogenous alkyne-Sph and its conversion to alkyne-Cer *via* the salvage pathway mediated by Cer synthase. This contrasts to the *de novo* synthesis pathway, where Cer is generated from serine and palmitoyl-coA *via* intermediates including dihydroceramide (66). While both the *de novo* and salvage Cer synthesis pathways are known to occur within cells, and the efficient conversion to alkyne-Cer and alkyne-SM clearly indicated efficient salvage of alkyne-Sph in our experiments (Figs. 2, *B* and *C* and 5*D*), it is possible that the effect of pUL21-mediated CERT activation could differ in magnitude for Cer synthesized *via* the *de novo* pathway. Future experiments could use radiolabeled serine to study how pUL21-mediated CERT activity affects the metabolism of Cer produced *via* the *de novo* pathway (67).

HSV-1 utilizes protein kinase D–mediated trafficking from the TGN to the plasma membrane (68). Disruption of protein kinase D–mediated trafficking has been shown to alter virus secretion, with siRNA-mediated depletion of CERT increasing the secretion of extracellular (cell-free) HSV-1 particles and CERT overexpression reducing virus secretion (38). We do not observe changes in the secretion of pUL21^V382E^ HSV-1, which lacks the ability to dephosphorylate CERT and thus activate CERT-mediated Cer transport. While these results may appear at first glance to be contradictory, it should be noted that in our experiments the cells contained WT levels of CERT, and cells infected with either ΔpUL21 or pUL21^V382E^ HSV-1 have similar levels of active (CERT^P^) and inactive (CERT^O^) protein compared with uninfected controls (Fig. 5*A*). In contrast, siRNA depletion would lead to a complete absence of CERT and of CERT-mediated lipid transfer. Furthermore, the previous study demonstrated that treatment of infected cells with HPA-12 did not stimulate virus particle secretion (38). This insensitivity of HSV-1 secretion to CERT pharmacological inhibition, combined with our observation that pUL21-mediated CERT activation does not alter HSV-1 secretion, strongly suggests that CERT catalytic activity is not directly linked to the regulation of virus secretion.

Our observation of labeled Cer accumulation during HSV-1 infection (Fig. 5) is consistent with an earlier report that HSV-1 infection causes an approximately twofold increase in Cer abundance within BHK-21 cells (34). Cer has many distinctive physical properties that set it apart from other membrane lipids: it has negative intrinsic curvature, it increases the order of phospholipids in membranes, and it makes biological membranes more permeable to even large solutes such as proteins (69). Nascent HSV-1 particles must traverse multiple biological membranes during virus assembly, most notably during capsid egress from the nucleus. Being too large to exit *via* nuclear pores, capsids leave by budding into and then out of the perinuclear space, the former step being catalyzed by the herpesvirus nuclear egress complex (NEC) (70). A recent study identified that the NEC induces lipid ordering to generate the negative curvature required for capsid budding (71). It is tempting to speculate that pUL21 regulates nuclear capsid egress by modulating Cer abundance, an effect that would be distinct from the previously demonstrated role of pUL21 in promoting nuclear egress *via* regulating the phosphorylation of NEC components (18, 20). In addition to potentially promoting virus nuclear egress, accumulation of Cer can lead to caspase 3 activation and apoptosis *via* increased mitochondrial outer membrane permeabilization (72). HSV-1 is known to encode proteins that defend against Cer-induced apoptosis (73), and it is possible that pUL21-stimulated acceleration of Cer to SM conversion serves to limit the proapoptotic activity of Cer, although confirmation of this requires further study.

In the process of probing the molecular basis of pUL21 binding to CERT, we serendipitously discovered a single amino acid substitution (L490E) that prevents pUL21 binding to HSV-1 pUL16 (Fig. 4*B*). Both pUL16 and pUL21 are known to facilitate efficient release of virus capsids from the nuclei of infected cells (74), and a recent study suggests that the pUL16–pUL21 interaction may help regulate capsid maturation and stability (75). Our mutagenesis is consistent with previous biochemical experiments that had mapped the binding of pUL16 to the C-terminal region of pUL21 (residues 268–535) and shown that the N-terminal 40 amino acids of pUL16 were dispensable for this interaction (10). Recent advances in deep learning structure prediction enable the accurate prediction of protein structures and complexes, even in challenging cases such as multidomain viral proteins with no known sequence homologs (76). We therefore generated a model of the pUL16–pUL21C interaction using AlphaFold-Multimer (52). pUL21 residue L490 lies at the center of the predicted interaction interface, consistent with its substitution to glutamate inhibiting pUL16 binding (Fig. 4, *B* and *H*). The structure of pUL16 has not been solved experimentally, and inspection of the pUL16 structural model provides two interesting observations. First, the closest structural homolog of the predicted pUL16 C-terminal domain is the N-terminal domain of pUL21 (Fig. 5*B* and Table S3). This structural correspondence (DALI Z-score 5.0; 3.6 Å rmsd across 121 C^α^ atoms) is striking given the low sequence identity (7%) shared by the domains, although it is known that viral proteins can share similar folds despite having unidentifiable sequence similarly (77). While identifiable pUL21 homologs are restricted to alphaherpesviruses (9), homologs of pUL16 can be found across *Herpesviridae* (78). The similarity of the predicted pUL16 C-terminal domain structure to that experimentally observed for the pUL21 N-terminal domain suggests that the two proteins are distant homologs, having arisen *via* a gene duplication event in an ancestral (alpha)herpesvirus. The second observation is that several proteins with structural similarity to the predicted pUL16 N-terminal domain have been associated with the transport of lipids or other bulky hydrophobic molecules (Fig. 5*D* and Table S3). For example, the VtrC protein from *Vibrio parahaemolyticus* was crystallized in complex with the large hydrophobic molecule taurodeoxycholate (79). Given the association between pUL21 and the lipid transport protein CERT, it is tempting to speculate that pUL16 may also have a role in modifying host or viral lipid membranes, but further biochemical and experimental structural analyses are required assess to whether the pUL16 N-terminal domain has any capacity to bind and/or transport lipids.

In summary, we demonstrated that pUL21 dephosphorylates and activates the cellular lipid transport protein CERT, stimulating conversion of Cer to SM. Characterizing the solution structure in complex with the PH and START domains of CERT allowed us to identify a single amino acid mutation of pUL21 that disrupts CERT dephosphorylation in infected cells. HSV-1 encoding this pUL21 mutant had similar replication kinetics, virus yields, and plaque sizes, confirming that dephosphorylation of other cellular and/or viral targets underpins the important role of pUL21 in HSV-1 replication and spread. The functional rationale for pUL21-mediated modulation of CERT activity by HSV-1 remains elusive, but we have defined the molecular tools that will allow its dissection in other cell types and/or animal models of infection.

## Experimental procedures

### Plasmids

The StrepII-CERT transient mammalian expression construct was described previously (18). For generation of stable cells, a synthetic gene encoding human CERT_L_ (UniProt ID: Q9Y5P4-1) was cloned into a modified version of plasmid PB-T (80) that encodes an N-terminal StrepII tag and a woodchuck hepatitis virus post-transcriptional regulatory element (81) in the 3′ untranslated region. The S132A substitution was introduced into this vector using QuikChange mutagenesis (Agilent) according to the manufacturer’s instructions. CERT_L_ destined for expression using *in vitro* transcription/translation system from wheat germ extract was cloned into the plasmid pF3A WG BYDV (Promega) with an N-terminal myc epitope tag. The truncations of CERT_L_ were generated by inverse PCR (MR, residues 123–364; START_L_, residues 365–624) or by introduction of a stop codon (PH, residues 1–122) using QuikChange mutagenesis. For purification following bacterial expression, miniCERT_L_ (residues 20–131 plus 351–624) was cloned into pOPTH (77), encoding an N-terminal MAH_6_ tag. The generation of pUL21-H_6_ and pUL21-GFP was described previously (18), and single amino acid substitutions were introduced by QuikChange mutagenesis. pUL21-H_6_ was subcloned into pOPTH, and inverse PCR was used to generate H_6_-pUL21C, encoding pUL21 amino acids 275 to 535. A plasmid (UK622) encoding mouse PP1γ (UniProt ID: P63087) with an N-terminal GST tag (82) was a kind gift from David Ron (Cambridge Institute for Medical Research). To generate pUL21^V382E^ HSV-1, pEPkan-S containing an I-SceI/KanR selection cassette was used (54).

### Mammalian cell culture

Mycoplasma-free spontaneously immortalized human keratinocyte (HaCaT) cells (83), HaCaT cells stably expressing pUL21 (HaCaT21) (18), African green monkey kidney (Vero) cells (American Type Culture Collection; catalog no.: CRL-1586), and HEK293T cells (American Type Culture Collection; catalog no.: CRL-3216) were maintained in Dulbecco’s modified Eagle's medium (DMEM) with high glucose (Merck), supplemented with 10% (v/v) heat-inactivated fetal calf serum and 2 mM l-glutamine (complete DMEM) in a humidified 5% CO_2_ atmosphere at 37 °C. For protein purification, Freestyle 293F suspension cells (Thermo Fisher Scientific) were grown in Freestyle 293F medium (Gibco) on a shaking platform (125 rpm) in a humidified 8% CO_2_ atmosphere at 37 °C.

Doxycycline-inducible stably transfected Freestyle 293F cells expressing StrepII-CERT_L_ and StrepII-CERT_L_^S132A^ were generated using a piggyBac transposon-based system (80). A 30 ml suspension culture of Freestyle 293F cells at 1 × 10^6^ cells/ml was transfected with a 5:1:1 mass ratio of PB-T-CERT_L_^(S132A)^:PB-RN:PBase (35 μg total DNA) using Freestyle MAX transfection reagent (Invitrogen) as per the manufacturer’s instructions. After 2 days, the cells were transferred to fresh media supplemented with 500 μg/ml geneticin (Gibco), and the drug selection was continued for 2 weeks with media replenishment every 3 days.

### GFP affinity capture

Monolayers of HEK293T cells grown in 9 cm dishes were transfected with TransIT-LT1 (Mirus) using 7.7 μg of pEGFP-N1 (for GFP alone), pUL21-GFP, or point mutants thereof, following the manufacturer’s instructions. At 24 h post-transfection, the cells were harvested by scraping into the medium, pelleted (220*g*, 5 min, 4 °C), washed three times with cold PBS, and lysed at 4 °C in 1 ml lysis buffer (10 mM Tris [pH 7.5], 150 mM NaCl, 0.5 mM EDTA, 0.5% NP-40, and 1:100 diluted EDTA-free protease inhibitor cocktail [Merck]) for 45 min before clarification (21,000*g*, 10 min, 4 °C). After immunoprecipitation with GFP-Trap beads (ChromoTek) performed in accordance with the manufacturer’s protocol, the samples were eluted by incubation at 95 °C for 5 min in 45 μl 2× SDS-PAGE loading buffer. Input and bound samples were separated by SDS-PAGE and analyzed by immunoblot.

### GST pulldown

GST pull-down experiments were carried out in 96-well flat-bottomed plates (Greiner) using magnetic glutathione beads (Thermo Fisher Scientific) to capture the GST-tagged pUL21 (bait protein). First, 0.5 nmol of purified bait protein was incubated with the beads at 4 °C for 30 min. The beads were washed three times with wash buffer (20 mM Tris [pH 8.5], 200 mM NaCl, 0.1% NP-40, 1 mM DTT, and 1 mM EDTA) and then incubated at 4 °C for 60 min with various truncations of myc-CERT_L_ (prey proteins), expressed using the TNT SP6 High Yield Wheat Germ *in vitro* transcription/translation system (Promega) in accordance with the manufacturer’s protocol, followed by four washes in wash buffer. Protein was eluted using wash buffer supplemented with 50 mM reduced glutathione before being analyzed by SDS-PAGE and immunoblotting.

### Antibodies

The following antibodies with listed dilutions were used for immunoblotting: rabbit anti-CERT (1:10,000 dilution; Abcam; catalog no.: ab72536), mouse anti-pUL21 (1:50 dilution; clone no.: 1F10-D12) (18) for Figure 1*F* (note: this monoclonal antibody does not recognize pUL21^V382E^), rabbit anti-pUL21 (1:5000 dilution (10)) for Figs. 5*A* and S6*A*, mouse anti-GAPDH (1:10,000 dilution; GeneTex; catalog no.: GTX28245), mouse anti-Myc (1:4000 dilution; Millipore; catalog no.: 05-724), mouse anti-PP1α (1:1000 dilution; Santa Cruz; catalog no.: sc-271762), rabbit anti-pUL16 (1:2000 dilution) (84), mouse anti-VP5 (1:50 dilution; catalog no.: DM165) (85), and rabbit anti-phospho-Akt substrates (1:1000 dilution; Cell Signalling; catalog no.: 9611). Fluorescently labeled secondary antibodies were used at 1:10,000 dilution: LI-COR IRDye 680T donkey anti-rabbit (catalog no.: 926-68023) and goat anti-mouse (catalog no.: 926-68020), LI-COR IRDye 800CW conjugated donkey anti-rabbit (catalog no.: 926-32213), and goat anti-mouse (catalog no.: 926-32210). For immunocytochemistry, mouse anti-pUL21 1:1 (clone no.: L1E4-C10), which was generated in the same immunization experiments as anti-pUL21 (clone no.: 1F10-D12) (18), and Alexa Fluor 488–conjugated goat anti-mouse (1:1000 dilution; Invitrogen; catalog no.: A21236) were used. For visualizing HSV-1 plaques, mouse anti-gD (LP2; 1:50 dilution) (86) and horseradish peroxidase–conjugated rabbit anti-mouse (1:5000 dilution; DaKo; catalog no.: P0161) were used.

### Recombinant protein purification following bacterial expression

All recombinant proteins were expressed in *Escherichia coli* T7 Express *lysY/I*^*q*^ cells (New England Biolabs). Except for GST-PP1γ, cells were grown in 2× TY medium at 37 °C to an absorbance at 600 nm of 0.8 to 1.2 before cooling to 22 °C and inducing protein expression by addition of 0.4 mM IPTG. At 16 to 20 h postinduction, cells were harvested by centrifugation, and pellets were stored at −70 °C until required. For GST-PP1γ, the 2× TY medium was supplemented with 1 mM MnCl_2_, and the cultures were cooled to 18 °C upon reaching an absorbance of 0.8 at 600 nm, followed by induction using 1 mM IPTG. For all recombinant proteins, cells were resuspended in lysis buffer (see later) at 4 °C before lysis using a TS series cell disruptor (Constant Systems) at 24 kpsi. Lysates were cleared by centrifugation (40,000*g*, 30 min, 4 °C) and incubated with the relevant affinity resins for 1 h at 4 °C before extensive washing (≥20 column volumes) and elution using the relevant elution buffer (see later). Samples were concentrated and subjected to SEC (see later). Fractions containing the desired protein as assessed by SDS-PAGE were pooled, concentrated, snap-frozen in liquid nitrogen, and stored at −70 °C.

The lysis buffer for pUL21-GST (20 mM Tris [pH 8.5], 300 mM NaCl, 0.5 mM MgCl_2_, 1.4 mM β-mercaptoethanol, and 0.05% Tween-20) and GST-PP1γ (50 mM Tris [pH 7.5], 500 mM NaCl, 1 mM MnCl_2_, 0.5 mM MgCl_2_, 1.4 mM β-mercaptoethanol, and 0.05% Tween-20) was supplemented with 200 to 400 U bovine DNase I (Merck) and 200 μl EDTA-free protease inhibitor cocktail (Merck). Cleared lysates were incubated with glutathione Sepharose 4B (Cytiva), washed with wash buffer (20 mM Tris [pH 8.5, pUL21-GST; pH 7.5, GST-PP1γ], 500 mM NaCl, 1 mM DTT, plus 1 mM MnCl_2_ [GST-PP1γ only]), and the proteins were eluted using wash buffer supplemented with 25 mM reduced glutathione. SEC was performed using a HiLoad Superdex 200 16/600 column (Cytiva) equilibrated in 20 mM Tris (pH 8.5), 500 mM NaCl, 1 mM DTT (pUL21-GST) or 50 mM Tris (pH 7.5), 100 mM NaCl, and 1 mM DTT (GST-PP1γ).

pUL21-H_6_, pUL21^V382E^-H_6_, and H_6_-pUL21C were purified in Tris buffer at pH 8.5 in 500 mM NaCl, and H_6_-miniCERT_L_ was purified in Tris buffer at pH 7.5 in 150 mM NaCl. Lysis buffer (20 mM Tris, 20 mM imidazole, NaCl, 0.5 mM MgCl_2_, 1.4 mM β-mercaptoethanol, and 0.05% Tween-20) was supplemented with 200 to 400 U bovine DNase I (Merck) and 200 μl EDTA-free protease inhibitor cocktail (Merck). Cleared lysates were incubated with nickel–nitrilotriacetic acid agarose (Qiagen), washed with wash buffer (20 mM Tris, 20 mM imidazole, and NaCl) and eluted using elution buffer (20 mM Tris, 250 mM imidazole, and NaCl). SEC was performed using a HiLoad Superdex 200 (pUL21-H_6_ and pUL21^V382E^-H_6_) or 75 (H_6_-pUL21C and H_6_-miniCERT_L_) 16/600 column equilibrated in 20 mM Tris, NaCl, and 1 mM DTT.

### Recombinant protein purification following mammalian cell expression

StrepII-CERT^P^ used for phosphatase assays was purified following transient transfection of Freestyle 293F cells, as described before (18). StrepII-CERT_L_^P^ and StrepII-CERT_L_^S132A^ were purified from stably transfected Freestyle 293F cells following induction with 2 μg/ml doxycycline (Fisher) for 72 h. Next, cells were harvested by centrifugation (220*g*, 5 min, 4 °C) and washed once with ice-cold PBS before being resuspended in ice-cold lysis buffer (with phosphatase inhibitors, StrepII-CERT_L_^P^ only) (100 mM Tris [pH 8.0], 150 mM NaCl, 0.5 mM EDTA, 1 mM DTT [10 mM tetrasodium pyrophosphate, 100 mM NaF, and 17.5 mM β-glycerophosphate]). Cells were lysed by passage through a 23G needle six times, and lysates were clarified by centrifugation (40,000*g*, 30 min, 4 °C). The supernatants were sonicated at 50% amplitude for 60 s using a sonicating probe (MSE), and the supernatants were passed through a 0.45 μm syringe filter (Sartorius). StrepII-tagged proteins were captured using 1 ml StrepTrap HP column (Cytiva) that had been pre-equilibrated in wash buffer (100 mM Tris [pH 8.0], 150 mM NaCl, 0.5 mM EDTA, and 1 mM DTT). After extensive washing (20 column volumes), the protein was eluted using wash buffer supplemented with 2.5 mM desthiobiotin. Pooled eluate was applied to a Superose 6 10/300 GL column (Cytiva) equilibrated in ITC buffer (20 mM Tris [pH 8.5], 500 mM NaCl, 0.5 mM Tris(2-carboxyethyl)phosphine [TCEP]), and fractions containing StrepII-CERT_L_ as assessed by SDS-PAGE were pooled, concentrated, and used for downstream applications.

### Mutagenesis of viral genomes and generation of recombinant HSV-1

All HSV-1 strain KOS viruses used in this study were reconstituted from a bacterial artificial chromosome (BAC) (87), and the mutated strain was generated using the two-step Red recombination method (54) with the following primers:

Forward: 5′-CGGCTCGTAGGCCGGTACACACAGCGCCACGGCCTGTACG**AA**CCTCGGCCCGACGACCCAGTAGGATGACGACGATAAGTAGGG.

Reverse: 5′-CGTTGATGGCATCGGCCAAGACTGGGTCGTCGGGCCGAGG**TT**CGTACAGGCCGTGGCGCTGTCAACCAATTAACCAATTCTGATTAG.

The generation of pUL21 deletion mutant (ΔpUL21) was described previously (18). To generate the P0 stocks, Vero cells were transfected with the recombinant BAC DNA together with pGS403 encoding Cre recombinase (to excise the BAC cassette) using TransIt-LT1 (Mirus) following the manufacturer’s instructions. After 3 days, the cells were scraped into the media, sonicated at 50% power for 30 s in a cup-horn sonicator (Branson), and titrated on Vero cell monolayers. The subsequent stocks were generated by infecting either Vero (HSV-1 WT) or HaCaT pUL21 cells (HSV-1 mutants) at an MOI of 0.01 for 3 days. The cells were then scraped and isolated by centrifugation at 1000*g* for 5 min. Pellets were resuspended in 1 ml of complete DMEM supplemented with 100 U/ml penicillin, 100 μg/ml streptomycin, and freeze/thawed thrice at −70 °C before being aliquoted, titered on Vero cell monolayers, and stored at −70 °C until required. The presence of the desired mutation in the reconstituted virus genomes was confirmed by sequencing the pUL21 gene.

### Metabolic labeling and lipid extraction for TLC

Metabolic labeling was performed using subconfluent (60–80% confluence) HaCaT or HaCaT21 cells grown in a 6-well plate. For analysis of stable cell lines, the cells were pretreated for 30 min with complete DMEM containing 1 μM *N*-(3-hydroxy-1-hydroxymethyl-3-phenylpropyl)dodecanamide (HPA-12; Tokyo Chemical Industry) dissolved in 0.1% dimethyl sulfoxide (DMSO; Merck), or 0.1% DMSO alone, and HPA-12 or DMSO was retained at the same concentrations throughout the subsequent incubation steps. For infection, cells were infected (see later) 14 h before metabolic labeling.

For metabolic labeling, cells were washed twice with warm PBS before incubation in 500 μl prewarmed DMEM with 1% (v/v) Nutridoma (Merck) supplemented with 5 μM alkyne-Sph (Cayman Chemical) for 5 min (pulse). Next, cells were washed twice with warm PBS, and 1 ml of prewarmed DMEM with 1% (v/v) Nutridoma was added to each well, followed by incubation at 37 °C for the indicated times of chase. Alkyne-Sph was stored as a 3.3 mM ethanolic stock solution at −20 °C.

At the indicated times of chase, the plate with cells was transferred onto the ice, washed twice with 1 ml ice-cold PBS, scraped into 300 μl of ice-cold PBS, and transferred into appropriate 1.5 ml microcentrifuge tubes containing 600 μl of methanol. To each tube, 150 μl of chloroform was added, followed by vigorous vortexing. The precipitated protein was pelleted (20,000*g*, 1 min) at room temperature (RT). The organic supernatant was transferred to separate 2 ml tubes containing 300 μl chloroform. About 600 μl of 0.1% acetic acid in water was subsequently added to each tube to induce formation of two phases. Following extensive vortexing, the phases were separated by centrifugation (20,000*g*, 1 min, RT), and the lower phase was transferred to a new 1.5 ml microcentrifuge tube. This lipid-containing solvent phase was dried in a UniVapo centrifugal vacuum concentrator (UniEquip) at 30 °C for 20 min.

If required for SDS-PAGE analysis, the protein pellet was solubilized with 5 μl 10% (w/v) SDS, diluted in 75 μl lysis buffer (20 mM Tris 7.5, 150 mM NaCl, and 1% [v/v] Triton X-100), boiled for 10 min, and sonicated at 75% power for 60 s in a cup-horn sonicator. The protein samples were mixed with 5× loading buffer before being subjected to SDS-PAGE analysis.

### Click reaction and TLC of labeled lipids

Lipid standards (16:0(alkyne)-18:1 PC [Avanti], pacFA GalCer [Avanti], d18:1(alkyne)-6:0 Cer [Cayman Chemicals], 18:0(alkyne) Sph [Cayman Chemicals]) or extracted lipids (aforementioned) were resuspended in 20 μl of 1:1 chloroform:ethanol. To each tube, 1 μl of Tris((1-benzyl-4-triazolyl)methyl)amine solution (2.5 mM in DMSO; Merck), 10 μl of Tetrakis(acetonitrile)copper(I) tetrafluoroborate (10 mM in acetonitrile; Merck), and 1 μl of 3-azido-7-hydroxycoumarin solution (1 mM in ethanol; Jena Bioscience) was added. After brief mixing, the reaction was dried in a UniVapo centrifugal vacuum concentrator (UniEquip) for 10 min at 45 °C. Lipids were dissolved in 20 μl of 65:25:4:1 chloroform:methanol:water:acetic acid, of which 8 μl was spotted on a 20 × 10 cm HPTLC Silica gel 60 plates (Merck). When dried, the plate was developed for 5 cm with 65:25:4:1 chloroform:methanol:water:acetic acid, dried again, and developed for 9 cm with 1:1 hexane:ethylacetate. Labeled lipids were visualized *via* UV illumination using a G:BOX Chemi XX9 (Syngene), bands were quantified using the Image Studio Lite software (LI-COR) with local background subtraction, and statistical tests were performed using Prism 7 (GraphPad Software, Inc).

### Virus infections

Monolayers of indicated cells were infected by overlaying with the appropriate viruses and diluted in complete DMEM to the specified MOI. The time of addition was designated 0 hpi. Cells were incubated with the inoculum at 37 °C in a humidified 5% CO_2_ atmosphere for 1 h, rocking the tissue culture plate housing the monolayers every 15 min, before complete DMEM supplemented with 100 U/ml penicillin, 100 μg/ml streptomycin was added to dilute the original infection medium fivefold. Infected cells were incubated at 37 °C in a humidified 5% CO_2_ atmosphere and harvested at the specified times.

### Immunoblotting

For analysis of protein expression and phosphorylation, cells were washed two times with ice-cold 50 mM Tris (pH 8.5), 150 mM NaCl, and scraped into 100 μl of ice-cold lysis buffer (50 mM Tris [pH 8.5], 150 mM NaCl, 1% [v/v] Triton X-100, 1% [v/v] EDTA-free protease inhibitor cocktail [Merck], 10 mM tetrasodium pyrophosphate, 100 mM NaF, and 17.5 mM β-glycerophosphate). After 30 min of incubation on ice, the lysates were sonicated (two 15 s pulses at 50% power) in a cup-horn sonicator followed by centrifugation at 20,000*g* for 10 min. For all samples, protein concentrations were determined using a bicinchoninic assay (Pierce), and normalized protein lysates were analyzed by SDS-PAGE. For enhanced separation of phosphorylated proteins, 7% (w/v) acrylamide gels contained 50 μM MnCl_2_ and 25 μM PhosTag reagent (Wako) where indicated. Separated proteins were then transferred to nitrocellulose membranes using the Mini-PROTEAN system (Bio-Rad) and analyzed by immunoblotting and signals being detected using Odyssey CLx Imaging System (LI-COR). For quantitation of CERT phosphoform relative abundance, the signal for CERT^O^ band alone and for all CERT bands (CERT^O^ + CERT^P^) were quantitated using Image Studio Lite software with local background subtraction. Statistical tests were performed using Prism 7.

### Analytical SEC of miniCERT_L_–pUL21C complex

Analytical SEC experiments were performed using Superdex 200 10/300 GL column equilibrated in 20 mM Tris (pH 8.5), 500 mM NaCl, and 1 mM DTT. To allow complex formation, 200 μl of 140 μM H_6_-miniCERT_L_ and 680 μl of 55.4 μM H_6_-pUL21C were mixed and incubated for 15 min at RT prior to injection.

### ITC

ITC experiments were performed at 25 °C using a MicroCal PEAQ-ITC automated calorimeter (Malvern Panalytical). Proteins were transferred into ITC buffer (20 mM Tris [pH 8.5], 500 mM NaCl, and 0.5 mM TCEP) either by SEC or extensive dialysis prior to experiments. Titrants (StrepII-CERT_L_^P^, StrepII-CERT_L_^S132A^, or H_6_-miniCERT_L_) were titrated into titrates (pUL21-H_6_, pUL21^V382E^-H_6_, or H_6_-pUL21C) using either 19 × 2 μl or 12 × 3 μl injections (Table S1). Data were analyzed using the MicroCal PEAQ-ITC analysis software (Malvern Panalytical) and fitted using a one-site binding model.

### MALS

MALS data were collected immediately following SEC (SEC–MALS) by inline measurement of static light scattering (DAWN 8+; Wyatt Technology), differential refractive index (Optilab T-rEX; Wyatt Technology), and UV absorbance (1260 UV; Agilent Technologies). Samples (100 μl) were injected onto an Superose 6 increase (all except H_6_-pUL21C) or Superdex 75 increase (H_6_-pUL21C) 10/300 GL columns equilibrated in 20 mM Tris (pH 8.5), 500 mM NaCl, and 0.5 mM TCEP at 0.4 ml/min. The protein samples were injected at the concentration of 2 mg/ml. Molecular masses were calculated using ASTRA 6 (Wyatt Technology) using a protein dn/dc of 0.186 ml/g.

### SAXS

SAXS experiments were performed in batch mode (H_6_-pUL21C and H_6_-miniCERT_L_) or inline following SEC (H_6_-pUL21C–H_6_-miniCERT_L_) at EMBL-P12 bioSAXS beamline (PETRAIII) (88, 89).

For SAXS in batch mode, scattering data (*I*(*s*) *versus s*, where *s* = 4πsinθ/λ nm^−1^, 2θ is the scattering angle, and λ is the X-ray wavelength, 0.124 nm) were collected from the indicated batch samples and a corresponding solvent blank (20 mM Tris [pH 8.5], 500 mM NaCl, 3% [v/v] glycerol, and 1 mM DTT). The scattering profiles of H_6_-miniCERT_L_ (1.73, 2.59, 3.46, and 6.91 mg/ml) and H_6_-pUL21C (0.76 mg/ml) were measured in continuous-flow mode using an automated sample changer (30 μl sample at 20 °C; 1 mm path length). The sample and buffer measurements were measured as 100 ms data frames on a Pilatus 6M area detector (Dectris) for total exposure times of 2.1 and 2.6 s, respectively. For H_6_-miniCERT_L_, the resulting SAXS curves were extrapolated to infinite dilution.

For SEC–SAXS experiments, the scattering data were recorded using a Pilatus 6M detector with 1 s sample exposure times for a total of 3600 data frames spanning the entire course of the SEC separation. Proteins were mixed at 1:1 molar ratio, incubated on ice for 15 min, and then concentrated using a centrifugal concentrator to the desired concentration as estimated from absorbance using extinction coefficients (90) calculated assuming a 1:1 molar ratio. About 40 μl of purified H_6_-pUL21C–H_6_-miniCERT_L_ (4.3 mg/ml) was injected at 0.35 ml/min onto a Superdex 200 Increase 5/150 GL column (Cytiva) equilibrated in 20 mM Tris (pH 8.5), 500 mM NaCl, 3% (v/v) glycerol, and 1 mM DTT. SAXS data were recorded from a single peak (frames 1245–1296 s), and solvent blank was collected from pre-elution frames (frames 247–411). Primary data reduction was carried out using CHROMIXS (91), and 2D-to-1D radial averaging was performed using the SASFLOW pipeline (92). Processing and analysis of the SAXS output was performed using the ATSAS 3.0.2 software package (93). The extrapolated forward scattering intensity at zero angle, *I*(0), and the radius of gyration, *R*_g_, were calculated from the Guinier approximation (ln*I*(*s*) *versus s*^2^, for *sR*_g_ < 1.3). The maximum particle dimension, *D*_max_, was estimated based on the probable distribution of real-space distances, *p*(*r*), which was calculated using GNOM (94). A concentration-independent estimate of molecular weight was determined using a Bayesian consensus method (95). All structural parameters are reported in Table S2.

*Ab initio* modeling was performed using GASBOR (49), and figures show the models that best fit their corresponding SAXS profiles (lowest χ^2^). Pseudoatomic modeling was performed using CORAL (96). For H_6_-miniCERT_L_, crystal structures of the CERT PH (Protein Data Bank [PDB] ID: 4HHV) (24) and START (PDB ID: 2E3M) (25) domains were used, the latter including predicted secondary structure as modeled by I-TASSER (97) for intron 11 (residues 371–396), present only in CERT_L_. The miniCERT_L_ 23 amino acid linker and the purification tag (MAH_6_) were modeled using CORAL. The model with the best fit was superimposed onto the PH:START crystal structure (PDB ID: 5JJD) (46) by aligning the START domains using PyMOL, version 2.5.2 (Schrödinger). For H_6_-pUL21C, the N-terminal (MAH_6_QD) and C-terminal (HGQSV) elements missing from the crystal structure (PDB ID: 5ED7) (51) were modeled using CORAL. For H_6_-pUL21C–H_6_-miniCERT_L_, the heterodimer was modeled using the SAXS curve of the complex using a fixed conformation of H_6_-pUL21C and mobile CERT domains, with unstructured elements modeled as aforementioned. All the models were assessed for fit to the corresponding SAXS profiles (χ^2^ and CorMap) using CRYSOL (98). Molecular images were generated using PyMOL.

### Sequence analysis, deep learning structure prediction, and structural analysis

Sequences of the C-terminal domain from pUL21 homologs were identified by querying the National Ceneter for Biotechnology Information nonredundant protein sequence database (99) with HSV-1 pUL21 (UniProt ID: F8RG07) residues 275 to 535 using BLASTP (100) for simplexviruses (taxid: 10294) and PSI-BLAST with two iterations for alphaherpesviruses (taxid: 10294). Truncated sequences (accession length <80% of HSV-1 pUL21) or those containing ambiguous amino acids (“X”) were manually removed. Sequences were aligned using COBALT (101) and are provided as supporting information (Data S1).

The structure of HSV-1 pUL16 (UniProt ID: P10200) in complex with pUL21C (residues 275–535, UniProt ID: F8RG07) was predicted using a locally installed version of AlphaFold-Multimer, version 2.2.2 (52). Structural similarity searches and superpositions were performed using the DALI web server (53). Molecular images were generated using PyMOL.

### Differential scanning fluorimetry

Differential scanning fluorimetry experiments were carried out using Viia7 real-time PCR system (Applied Biosystems) and 1× Protein Thermal Shift dye (Applied Biosystems). The assay buffer (20 mM Tris [pH 8.5], 500 mM NaCl, and 1 mM DTT) was mixed with dye stock solution and protein solution (H_6_-pUL21 or H_6_-pUL21^V382E^) in an 8:1:1 ratio, giving 1 ng protein in a final volume of 20 μl, and measurements were performed in triplicate. Samples were heated from 25 to 95 °C at 1° per 20 s, and fluorescence was monitored at each increment. The melting temperature (*T*_m_) is the inflection point of the sigmoidal melting curve, determined by nonlinear curve fitting to the Boltzmann equation using Prism 7.

### *In vitro* dephosphorylation assays

Dephosphorylation assays were performed upon 0.5 μM of purified CERT^P^ using a fixed concentration of GST-PP1γ (25 nM) in the presence of H_6-_pUL21 or H_6_-pUL21^V382E^ at the indicated concentrations. Reactions (50 μl) proceeded in assay buffer (150 mM NaCl, 20 mM Tris [pH 8.5], 0.1% Tween-20, and 1 mM MnCl_2_) for 30 min at 30 °C before being stopped by the addition of 50 μl of 2× SDS-PAGE loading buffer and boiling at 95 °C for 5 min. Samples were analyzed by SDS-PAGE using 7% (w/v) acrylamide gels supplemented with 25 μM PhosTag reagent and 50 μM MnCl_2_, and the protein was visualized using InstantBlue Coomassie stain (Expedeon). To measure the ratio of CERT^O^ to total CERT, Coomassie-stained gels were scanned using an Odyssey CLx Imaging System (LI-COR). The signals detected in the 700 nm channel for the CERT^O^ band alone and for all CERT bands (CERT^O^ + CERT^P^) were quantitated using Image Studio Lite software with local background subtraction.

### Plaque size analysis

Confluent monolayers of Vero or HaCaT cells in 6-well tissue culture plates were infected with 100 plaque-forming units of the indicated virus diluted in complete DMEM to a final volume of 500 μl. Following the adsorption for 1 h at 37 °C in a humidified 5% CO_2_ atmosphere, rocking the plate every 15 min, the cells were overlaid with plaque assay media (DMEM supplemented with 0.3% high viscosity carboxymethyl cellulose, 0.3% low viscosity carboxymethyl cellulose, 2% [v/v] fetal calf serum, 2 mM l-glutamine, 100 U/ml penicillin, and 100 μg/ml streptomycin) and incubated for further 48 h. Next, cells were fixed with 3.7% (v/v) formal saline for 20 min, washed three times with PBS, and incubated for 1 h with mouse anti-gD (LP2) (86), diluted 1:50 in blocking buffer (PBS supplemented with 1% [w/v] bovine serum albumin and 0.1% Tween-20). Cells were washed thrice in PBS and incubated for 30 min with horseradish peroxidase–conjugated rabbit anti-mouse antibody (DaKo; catalog no.: P0161) diluted 1:5000 in blocking buffer, followed by two subsequent PBS washes and one wash with ultrapure water. Plaques were visualized using the TrueBlue peroxidase substrate in accordance with the manufacturer’s instructions (Seracare). Plaques were scanned at 1200 dpi, and plaque areas were measured using Fiji (102, 103).

### Single-step (high MOI) virus growth assays

Virus growth assays were performed in technical duplicate for each independent experiment. Monolayers of HaCaT or Vero cells were infected with the indicated viruses diluted in complete DMEM to an MOI of 10. The time of virus addition was designated 0 hpi. After adsorption for 1 h at 37 °C in a humidified 5% CO_2_ atmosphere, rocking the plates every 15 min, extracellular virus particles were neutralized with an acid wash (40 mM citric acid, 135 mM NaCl, 10 mM KCl, pH 3.0) for 1 min, and cells were then washed three times with PBS before being subsequently overlaid with complete DMEM. At designated times postinfection, cells were harvested by freezing the plate at −70 °C. When all plates were frozen, samples were freeze/thawed one more time before scraping and transferring to 1.5 ml tubes and storing at −70 °C until titration. Titrations were performed on monolayers of Vero cells. Serial dilutions of the samples were used to inoculate the cells for 1 h, followed by overlaying with DMEM containing 0.3% high-viscosity carboxymethyl cellulose, 0.3% low-viscosity carboxymethyl cellulose, 2% (v/v) fetal bovine seum, 2 mM l-glutamine, 100 U/ml penicillin, and 100 μg/ml streptomycin. Three days later, cells were fixed in 3.7% (v/v) formal saline for 20 min, washed with water, and stained with 0.1% toluidine blue. Statistical tests were performed using Prism 7.

### Virus release assays

Monolayers of HaCaT or Vero cells were infected as described previously for single-step virus growth assays, infections being performed in technical duplicate or triplicate for each independent experiment. At 12 hpi, the media (500 μl) were harvested to 1.5 ml Eppendorf tubes, centrifuged for 5 min at 1000*g* to remove any detached cells, and 300 μl of the supernatant was carefully transferred to fresh tubes and stored at −70 °C until titration. The cells were overlaid with 500 μl of fresh DMEM, and the plates were immediately frozen at −70 °C. Titrations were performed on monolayers of Vero cells as described previously. Statistical tests were performed using Prism 7. Figure 6*C* was generated using SuperPlotsOfData (104).

### Immunocytochemistry

Cells grown on #1.5 coverslips were infected at an MOI of 1 as described previously. At 14 hpi, cells were washed with PBS and incubated with freezing cold (−20 °C) methanol for 5 min at −20 °C. Coverslips were washed with PBS, followed by incubation with blocking buffer (1% [w/v] bovine serum albumin in PBS) for 30 min at RT. Primary antibodies (aforementioned) were diluted in blocking buffer and incubated with coverslips for 1 h. Coverslips were washed 10 times with PBS before incubation for 45 min with the secondary antibodies (aforementioned) diluted in blocking buffer. Coverslips were washed 10 times in PBS and 10 times in ultrapure water before mounting on slides using Mowiol 4-88 (Merck) containing 200 nM 4′,6-diamidino-2-phenylindole. Images were acquired using an inverted Olympus IX81 widefield microscope with a 60× Plan Apochromat N oil objective (numerical aperture of 1.42) (Olympus) and Retiga EXi Fast1394 interline CCD camera (QImaging).

## Data availability

The SAXS data measured for each concentration, with an accompanying report, are available in the Small Angle Scattering Biological Data Bank (SASBDB) (105), entries SASDNB7, SASDNC7, and SASDND7. The AlphaFold-Multimer model of the HSV-1 pUL16–pUL21C complex, with associated raw output and quality statistics, has been deposited in the University of Cambridge Data Repository (https://doi.org/10.17863/CAM.88729).

## Supporting information

This article contains supporting information (18, 50, 51, 53, 79, 92, 106, 107, 108, 109, 110, 111).

## Conflict of interest

The authors declare that they have no conflicts of interest with the contents of this article.

## References

[1] Looker K.J., Magaret A.S., May M.T., Turner K.M.E., Vickerman P., Gottlieb S.L. (2015). Global and regional estimates of prevalent and incident herpes simplex virus type 1 infections in 2012. PLoS One.

[2] Kulej K., Avgousti D.C., Sidoli S., Herrmann C., Fera A.N.D., Kim E.T. (2017). Time-resolved global and chromatin proteomics during herpes simplex virus type 1 (HSV-1) infection. Mol. Cell. Proteomics.

[3] Soh T.K., Davies C.T.R., Muenzner J., Hunter L.M., Barrow H.G., Connor V. (2020). Temporal proteomic analysis of herpes simplex virus 1 infection reveals cell-surface remodeling via pUL56-mediated GOPC degradation. Cell Rep..

[4] Scherer K.M., Manton J.D., Soh T.K., Mascheroni L., Connor V., Crump C.M. (2021). A fluorescent reporter system enables spatiotemporal analysis of host cell modification during herpes simplex virus-1 replication. J. Biol. Chem..

[5] Nahas K.L., Connor V., Scherer K.M., Kaminski C.F., Harkiolaki M., Crump C.M. (2022). Near-native state imaging by cryo-soft-X-ray tomography reveals remodelling of multiple cellular organelles during HSV-1 infection. PLoS Pathog..

[6] Bigalke J.M., Heldwein E.E. (2016). Nuclear exodus: herpesviruses lead the way. Annu. Rev. Virol..

[7] Owen D.J., Crump C.M., Graham S.C. (2015). Tegument assembly and secondary envelopment of alphaherpesviruses. Viruses.

[8] Cocchi F., Menotti L., Dubreuil P., Lopez M., Campadelli-Fiume G. (2000). Cell-to-cell spread of wild-type herpes simplex virus type 1, but not of syncytial strains, is mediated by the immunoglobulin-like receptors that mediate virion entry, Nectin1 (PRR1/HveC/HIgR) and Nectin2 (PRR2/HveB). J. Virol..

[9] Le Sage V., Jung M., Alter J.D., Wills E.G., Johnston S.M., Kawaguchi Y. (2013). The herpes simplex virus 2 UL21 protein is essential for virus propagation. J. Virol..

[10] Harper A.L., Meckes D.G., Marsh J.A., Ward M.D., Yeh P.-C., Baird N.L. (2010). Interaction domains of the UL16 and UL21 tegument proteins of herpes simplex virus. J. Virol..

[11] Klupp B.G., Böttcher S., Granzow H., Kopp M., Mettenleiter T.C. (2005). Complex formation between the UL16 and UL21 tegument proteins of pseudorabies virus. J. Virol..

[12] Han J., Chadha P., Starkey J.L., Wills J.W. (2012). Function of glycoprotein E of herpes simplex virus requires coordinated assembly of three tegument proteins on its cytoplasmic tail. Proc. Natl. Acad. Sci. U. S. A..

[13] Takakuwa H., Goshima F., Koshizuka T., Murata T., Daikoku T., Nishiyama Y. (2001). Herpes simplex virus encodes a virion-associated protein which promotes long cellular processes in over-expressing cells. Genes Cells.

[14] Yan K., Liu J., Guan X., Yin Y.-X., Peng H., Chen H.-C. (2019). The carboxyl terminus of tegument protein pUL21 contributes to pseudorabies virus neuroinvasion. J. Virol..

[15] Finnen R.L., Banfield B.W. (2018). CRISPR/Cas9 mutagenesis of UL21 in multiple strains of herpes simplex virus reveals differential requirements for pUL21 in viral replication. Viruses.

[16] Sarfo A., Starkey J., Mellinger E., Zhang D., Chadha P., Carmichael J. (2017). The UL21 tegument protein of herpes simplex virus 1 is differentially required for the syncytial phenotype. J. Virol..

[17] Klupp B.G., Lomniczi B., Visser N., Fuchs W., Mettenleiter T.C. (1995). Mutations affecting the UL21 gene contribute to avirulence of pseudorabies virus vaccine strain Bartha. Virology.

[18] Benedyk T.H., Muenzner J., Connor V., Han Y., Brown K., Wijesinghe K.J. (2021). pUL21 is a viral phosphatase adaptor that promotes herpes simplex virus replication and spread. PLoS Pathog..

[19] Peti W., Nairn A.C., Page R. (2013). Structural basis for protein phosphatase 1 regulation and specificity. FEBS J..

[20] Muradov J.H., Finnen R.L., Gulak M.A., Hay T.J.M., Banfield B.W. (2021). pUL21 regulation of pUs3 kinase activity influences the nature of nuclear envelope deformation by the HSV-2 nuclear egress complex. PLoS Pathog..

[21] Fukasawa M., Nishijima M., Hanada K. (1999). Genetic evidence for ATP-dependent endoplasmic reticulum-to-Golgi apparatus trafficking of ceramide for sphingomyelin synthesis in Chinese hamster ovary cells. J. Cell Biol..

[22] Funakoshi T., Yasuda S., Fukasawa M., Nishijima M., Hanada K. (2000). Reconstitution of ATP- and cytosol-dependent transport of de novo synthesized ceramide to the site of sphingomyelin synthesis in semi-intact cells. J. Biol. Chem..

[23] Hanada K., Kumagai K., Yasuda S., Miura Y., Kawano M., Fukasawa M. (2003). Molecular machinery for non-vesicular trafficking of ceramide. Nature.

[24] Prashek J., Truong T., Yao X. (2013). Crystal structure of the pleckstrin homology domain from the ceramide transfer protein: implications for conformational change upon ligand binding. PLoS One.

[25] Kudo N., Kumagai K., Tomishige N., Yamaji T., Wakatsuki S., Nishijima M. (2008). Structural basis for specific lipid recognition by CERT responsible for nonvesicular trafficking of ceramide. Proc. Natl. Acad. Sci. U. S. A..

[26] Charruyer A., Bell S.M., Kawano M., Douangpanya S., Yen T.-Y., Macher B.A. (2008). Decreased ceramide transport protein (CERT) function alters sphingomyelin production following UVB irradiation. J. Biol. Chem..

[27] Kawano M., Kumagai K., Nishijima M., Hanada K. (2006). Efficient trafficking of ceramide from the endoplasmic reticulum to the Golgi apparatus requires a VAMP-associated protein-interacting FFAT motif of CERT. J. Biol. Chem..

[28] Kumagai K., Kawano M., Shinkai-Ouchi F., Nishijima M., Hanada K. (2007). Interorganelle trafficking of ceramide is regulated by phosphorylation-dependent cooperativity between the PH and START domains of CERT. J. Biol. Chem..

[29] Hannun Y.A., Obeid L.M. (2018). Sphingolipids and their metabolism in physiology and disease. Nat. Rev. Mol. Cell Biol..

[30] Sezgin E., Levental I., Mayor S., Eggeling C. (2017). The mystery of membrane organization: composition, regulation and roles of lipid rafts. Nat. Rev. Mol. Cell Biol..

[31] Olsen A.S.B., Faergeman N.J. (2017). Sphingolipids: membrane microdomains in brain development, function and neurological diseases. Open Biol..

[32] Elwell C.A., Engel J.N. (2012). Lipid acquisition by intracellular Chlamydiae. Cell. Microbiol..

[33] Gewaid H., Aoyagi H., Arita M., Watashi K., Suzuki R., Sakai S. (2020). Sphingomyelin is essential for the structure and function of the double-membrane vesicles in hepatitis C virus RNA replication factories. J. Virol..

[34] Ray E.K., Blough H.A. (1978). The effect of herpesvirus infection and 2-deoxy-d-glucose on glycosphingolipids in BHK-21 cells. Virology.

[35] Steinhart W.L., Busch J.S., Oettgen J.P., Howland J.L. (1984). Sphingolipid metabolism during infection of human fibroblasts by herpes simplex virus type 1. Intervirology.

[36] Pastenkos G., Miller J.L., Pritchard S.M., Nicola A.V. (2019). Role of sphingomyelin in alphaherpesvirus entry. J. Virol..

[37] Lang J., Bohn P., Bhat H., Jastrow H., Walkenfort B., Cansiz F. (2020). Acid ceramidase of macrophages traps herpes simplex virus in multivesicular bodies and protects from severe disease. Nat. Commun..

[38] Roussel E., Lippe R. (2018). Cellular protein kinase D modulators play a role during multiple steps of herpes simplex virus 1 egress. J. Virol..

[39] Shevchenko A., Simons K. (2010). Lipidomics: coming to grips with lipid diversity. Nat. Rev. Mol. Cell Biol..

[40] Sunshine H., Iruela-Arispe M.L. (2017). Membrane lipids and cell signaling. Curr. Opin. Lipidol..

[41] Gaebler A., Milan R., Straub L., Hoelper D., Kuerschner L., Thiele C. (2013). Alkyne lipids as substrates for click chemistry-based *in vitro* enzymatic assays. J. Lipid Res..

[42] Gerl M.J., Bittl V., Kirchner S., Sachsenheimer T., Brunner H.L., Lüchtenborg C. (2016). Sphingosine-1-phosphate lyase deficient cells as a tool to study protein lipid interactions. PLoS One.

[43] Morash S.C., Cook H.W., Spence M.W. (1989). Lysophosphatidylcholine as an intermediate in phosphatidylcholine metabolism and glycerophosphocholine synthesis in cultured cells: an evaluation of the roles of 1-acyl- and 2-acyl-lysophosphatidylcholine. Biochim. Biophys. Acta.

[44] Yasuda S., Kitagawa H., Ueno M., Ishitani H., Fukasawa M., Nishijima M. (2001). A novel inhibitor of ceramide trafficking from the endoplasmic reticulum to the site of sphingomyelin synthesis. J. Biol. Chem..

[45] Prischi F., Filippakopoulos P. (2021). Editorial: structural studies of protein complexes in signaling pathways. Front. Mol. Biosci..

[46] Prashek J., Bouyain S., Fu M., Li Y., Berkes D., Yao X. (2017). Interaction between the PH and START domains of ceramide transfer protein competes with phosphatidylinositol 4-phosphate binding by the PH domain. J. Biol. Chem..

[47] Sugiki T., Takeuchi K., Yamaji T., Takano T., Tokunaga Y., Kumagai K. (2012). Structural basis for the Golgi association by the pleckstrin homology domain of the ceramide trafficking protein (CERT). J. Biol. Chem..

[48] Raya A., Revert-Ros F., Martinez-Martinez P., Navarro S., Roselló E., Vieites B. (2000). Goodpasture antigen-binding protein, the kinase that phosphorylates the goodpasture antigen, is an alternatively spliced variant implicated in autoimmune pathogenesis. J. Biol. Chem..

[49] Svergun D.I., Petoukhov M.V., Koch M.H.J. (2001). Determination of domain structure of proteins from X-ray solution scattering. Biophys. J..

[50] Metrick C.M., Chadha P., Heldwein E.E. (2015). The unusual fold of herpes simplex virus 1 UL21, a multifunctional tegument protein. J. Virol..

[51] Metrick C.M., Heldwein E.E. (2016). Novel structure and unexpected RNA-binding ability of the C-terminal domain of herpes simplex virus 1 tegument protein UL21. J. Virol..

[52] Evans R., O'Neill M., Pritzel A., Antropova N., Senior A., Green T. (2021). Protein complex prediction with alphafold-multimer. bioRxiv.

[53] Holm L. (2022). Dali server: structural unification of protein families. Nucleic Acids Res..

[54] Tischer B.K., Smith G.A., Osterrieder N. (2010). En passant mutagenesis: a two step markerless red recombination system. Methods Mol. Biol..

[55] Chuluunbaatar U., Roller R., Feldman M.E., Brown S., Shokat K.M., Mohr I. (2010). Constitutive mTORC1 activation by a herpesvirus Akt surrogate stimulates mRNA translation and viral replication. Genes Dev..

[56] Bell C., Desjardins M., Thibault P., Radtke K. (2013). Proteomics analysis of herpes simplex virus type 1-infected cells reveals dynamic changes of viral protein expression, ubiquitylation, and phosphorylation. J. Proteome Res..

[57] Langeland N., Haarr L., Holmsen H. (1986). Polyphosphoinositide metabolism in baby-hamster kidney cells infected with herpes simplex virus type 1. Biochem. J..

[58] Sutter E., de Oliveira A.P., Tobler K., Schraner E.M., Sonda S., Kaech A. (2012). Herpes simplex virus 1 induces de novo phospholipid synthesis. Virology.

[59] Wargo A.R., Kurath G. (2012). Viral fitness: definitions, measurement, and current insights. Curr. Opin. Virol..

[60] Han X. (2016). Lipidomics for studying metabolism. Nat. Rev. Endocrinol..

[61] Xu T., Hu C., Xuan Q., Xu G. (2020). Recent advances in analytical strategies for mass spectrometry-based lipidomics. Anal. Chim. Acta.

[62] Campadelli G., Brandimarti R., Lazzaro C.D., Ward P.L., Roizman B., Torrisi M.R. (1993). Fragmentation and dispersal of Golgi proteins and redistribution of glycoproteins and glycolipids processed through the Golgi apparatus after infection with herpes simplex virus 1. Proc. Natl. Acad. Sci. U. S. A..

[63] Kumagai K., Hanada K. (2019). Structure, functions and regulation of CERT, a lipid-transfer protein for the delivery of ceramide at the ER-Golgi membrane contact sites. FEBS Lett..

[64] Jain A., Beutel O., Ebell K., Korneev S., Holthuis J.C.M. (2017). Diverting CERT-mediated ceramide transport to mitochondria triggers Bax-dependent apoptosis. J. Cell Sci..

[65] Bockelmann S., Mina J.G.M., Korneev S., Hassan D.G., Müller D., Hilderink A. (2018). A search for ceramide binding proteins using bifunctional lipid analogs yields CERT-related protein StarD7. J. Lipid Res..

[66] Kitatani K., Idkowiak-Baldys J., Hannun Y.A. (2008). The sphingolipid salvage pathway in ceramide metabolism and signaling. Cell Signal..

[67] Hanada K., Nishijima M. (2000). Selection of mammalian cell mutants in sphingolipid biosynthesis. Methods Enzymol..

[68] Rémillard-Labrosse G., Mihai C., Duron J., Guay G., Lippé R. (2009). Protein kinase D-dependent trafficking of the large herpes simplex virus type 1 capsids from the TGN to plasma membrane. Traffic.

[69] Alonso A., Goñi F.M. (2018). The physical properties of ceramides in membranes. Annu. Rev. Biophys..

[70] Bigalke J.M., Heldwein E.E. (2015). Structural basis of membrane budding by the nuclear egress complex of herpesviruses. EMBO J..

[71] Thorsen M.K., Lai A., Lee M.W., Hoogerheide D.P., Wong G.C.L., Freed J.H. (2021). Highly basic clusters in the herpes simplex virus 1 nuclear egress complex drive membrane budding by inducing lipid ordering. mBio.

[72] Ogretmen B. (2018). Sphingolipid metabolism in cancer signalling and therapy. Nat. Rev. Cancer.

[73] Galvan V., Roizman B. (1998). Herpes simplex virus 1 induces and blocks apoptosis at multiple steps during infection and protects cells from exogenous inducers in a cell-type-dependent manner. Proc. Natl. Acad. Sci. U. S. A..

[74] Gao J., Finnen R.L., Sherry M.R., Le Sage V., Banfield B.W. (2020). Differentiating the roles of UL16, UL21, and Us3 in the nuclear egress of herpes simplex virus capsids. J. Virol..

[75] Thomas E.C.M., Bossert M., Banfield B.W. (2022). The herpes simplex virus tegument protein pUL21 is required for viral genome retention within capsids. bioRxiv.

[76] Gao W.N.D., Gao C., Deane J.E., Carpentier D.C.J., Smith G.L., Graham S.C. (2022). The crystal structure of vaccinia virus protein E2 and perspectives on the prediction of novel viral protein folds. J. Gen. Virol..

[77] Neidel S., Maluquer de Motes C., Mansur D.S., Strnadova P., Smith G.L., Graham S.C. (2015). Vaccinia virus protein A49 is an unexpected member of the B-cell lymphoma (Bcl)-2 protein family. J. Biol. Chem..

[78] Wing B.A., Lee G.C., Huang E.S. (1996). The human cytomegalovirus UL94 open reading frame encodes a conserved herpesvirus capsid/tegument-associated virion protein that is expressed with true late kinetics. J. Virol..

[79] Li P., Rivera-Cancel G., Kinch L.N., Salomon D., Tomchick D.R., Grishin N.V. (2016). Bile salt receptor complex activates a pathogenic type III secretion system. Elife.

[80] Li Z., Michael I.P., Zhou D., Nagy A., Rini J.M. (2013). Simple piggy Bac transposon-based mammalian cell expression system for inducible protein production. Proc. Natl. Acad. Sci. U. S. A..

[81] Zufferey R., Donello J.E., Trono D., Hope T.J. (1999). Woodchuck hepatitis virus posttranscriptional regulatory element enhances expression of transgenes delivered by retroviral vectors. J. Virol..

[82] Chen R., Rato C., Yan Y., Crespillo-Casado A., Clarke H.J., Harding H.P. (2015). G-actin provides substrate-specificity to eukaryotic initiation factor 2α holophosphatases. Elife.

[83] Boukamp P., Petrussevska R.T., Breitkreutz D., Hornung J., Markham A., Fusenig N.E. (1988). Normal keratinization in a spontaneously immortalized aneuploid human keratinocyte cell line. J. Cell Biol..

[84] Carmichael J.C., Wills J.W. (2019). Differential requirements for gE, gI, and UL16 among herpes simplex virus 1 syncytial variants suggest unique modes of dysregulating the mechanism of cell-to-cell spread. J. Virol..

[85] McClelland D.A., Aitken J.D., Bhella D., McNab D., Mitchell J., Kelly S.M. (2002). pH reduction as a trigger for dissociation of herpes simplex virus type 1 scaffolds. J. Virol..

[86] Minson A.C., Hodgman T.C., Digard P., Hancock D.C., Bell S.E., Buckmaster E.A. (1986). An analysis of the biological properties of monoclonal antibodies against glycoprotein D of herpes simplex virus and identification of amino acid substitutions that confer resistance to neutralization. J. Gen. Virol..

[87] Gierasch W.W., Zimmerman D.L., Ward S.L., VanHeyningen T.K., Romine J.D., Leib D.A. (2006). Construction and characterization of bacterial artificial chromosomes containing HSV-1 strains 17 and KOS. J. Virol. Methods.

[88] Blanchet C.E., Spilotros A., Schwemmer F., Graewert M.A., Kikhney A., Jeffries C.M. (2015). Versatile sample environments and automation for biological solution X-ray scattering experiments at the P12 beamline (PETRA III, DESY). J. Appl. Crystallogr..

[89] Graewert M.A., Franke D., Jeffries C.M., Blanchet C.E., Ruskule D., Kuhle K. (2015). Automated pipeline for purification, biophysical and x-ray analysis of biomacromolecular solutions. Sci. Rep..

[90] Wilkins M.R., Gasteiger E., Bairoch A., Sanchez J.C., Williams K.L., Appel R.D. (1999). Protein identification and analysis tools in the ExPASy server. Methods Mol. Biol..

[91] Panjkovich A., Svergun D.I. (2018). CHROMIXS: automatic and interactive analysis of chromatography-coupled small-angle X-ray scattering data. Bioinformatics.

[92] Franke D., Jeffries C.M., Svergun D.I. (2015). Correlation map, a goodness-of-fit test for one-dimensional X-ray scattering spectra. Nat. Methods.

[93] Manalastas-Cantos K., Konarev P.V., Hajizadeh N.R., Kikhney A.G., Petoukhov M.V., Molodenskiy D.S. (2021). Atsas 3.0: expanded functionality and new tools for small-angle scattering data analysis. J. Appl. Crystallogr..

[94] Svergun D.I. (1992). Determination of the regularization parameter in indirect-transform methods using perceptual criteria. J. Appl. Crystallogr..

[95] Hajizadeh N.R., Franke D., Jeffries C.M., Svergun D.I. (2018). Consensus Bayesian assessment of protein molecular mass from solution X-ray scattering data. Sci. Rep..

[96] Petoukhov M.V., Franke D., Shkumatov A.V., Tria G., Kikhney A.G., Gajda M. (2012). New developments in the ATSAS program package for small-angle scattering data analysis. J. Appl. Crystallogr..

[97] Yang J., Yan R., Roy A., Xu D., Poisson J., Zhang Y. (2015). The I-tasser suite: protein structure and function prediction. Nat. Methods.

[98] Svergun D., Barberato C., Koch M.H.J. (1995). *Crysol* – a program to evaluate X-ray solution scattering of biological macromolecules from atomic coordinates. J. Appl. Crystallogr..

[99] Sayers E.W., Bolton E.E., Brister J.R., Canese K., Chan J., Comeau D.C. (2022). Database resources of the national center for biotechnology information. Nucleic Acids Res..

[100] Altschul S.F., Gish W., Miller W., Myers E.W., Lipman D.J. (1990). Basic local alignment search tool. J. Mol. Biol..

[101] Papadopoulos J.S., Agarwala R. (2007). Cobalt: constraint-based alignment tool for multiple protein sequences. Bioinformatics.

[102] Rueden C.T., Schindelin J., Hiner M.C., DeZonia B.E., Walter A.E., Arena E.T. (2017). ImageJ2: ImageJ for the next generation of scientific image data. BMC Bioinformatics.

[103] Schindelin J., Arganda-Carreras I., Frise E., Kaynig V., Longair M., Pietzsch T. (2012). Fiji: an open-source platform for biological-image analysis. Nat. Methods.

[104] Goedhart J. (2021). SuperPlotsOfData-a web app for the transparent display and quantitative comparison of continuous data from different conditions. Mol. Biol. Cell.

[105] Valentini E., Kikhney A.G., Previtali G., Jeffries C.M., Svergun D.I. (2015). SASBDB, a repository for biological small-angle scattering data. Nucleic Acids Res..

[106] Crooks G.E., Hon G., Chandonia J.-M., Brenner S.E. (2004). WebLogo: a sequence logo generator. Genome Res..

[107] Wang Z., Shen H., He B., Teng M., Guo Q., Li X. (2021). The structural mechanism for the nucleoside tri- and diphosphate hydrolysis activity of Ntdp from Staphylococcus aureus. FEBS J..

[108] Miliara X., Garnett J.A., Tatsuta T., Abid Ali F., Baldie H., Pérez-Dorado I. (2015). Structural insight into the TRIAP1/PRELI-like domain family of mitochondrial phospholipid transfer complexes. EMBO Rep..

[109] Kryshtafovych A., Albrecht R., Baslé A., Bule P., Caputo A.T., Carvalho A.L. (2018). Target highlights from the first post-PSI CASP experiment (CASP12, May-August 2016). Proteins.

[110] Hirano H., Gootenberg J.S., Horii T., Abudayyeh O.O., Kimura M., Hsu P.D. (2016). Structure and engineering of francisella novicida Cas9. Cell.

[111] Chekan J.R., Koos J.D., Zong C., Maksimov M.O., Link A.J., Nair S.K. (2016). Structure of the lasso peptide isopeptidase identifies a topology for processing threaded substrates. J. Am. Chem. Soc..

[112] Wakashima T., Abe K., Kihara A. (2014). Dual functions of the trans-2-enoyl-CoA reductase TER in the sphingosine 1-phosphate metabolic pathway and in fatty acid elongation. J. Biol. Chem..

[113] Coleman R.A., Lee D.P. (2004). Enzymes of triacylglycerol synthesis and their regulation. Prog. Lipid Res..

[114] Receveur-Brechot V., Durand D. (2012). How random are intrinsically disordered proteins? A small angle scattering perspective. Curr. Protein Pept. Sci..

