# Supplementary material for: Herpes simplex virus 1 protein pUL21 alters ceramide metabolism by activating the interorganelle transport protein CERT
Source: J Biol Chem. 2022 Oct 13;298(11):102589. doi: 10.1016/j.jbc.2022.102589 (PMC9668737; doi:10.1016/j.jbc.2022.102589)
Supplement: Supplementary Material [file mmc1.pdf]

Supporting Information

Herpes simplex virus 1 protein pUL21 alters ceramide metabolism by activating the inter-organelle transport protein CERT

Tomasz H. Benedyk[1,a], Viv Connor[1], Eve R. Caroe[2,b], Maria Shamin[2], Dmitri I. Svergun[3], Janet E. Deane[2], Cy M. Jeffries[3], Colin M. Crump[1], Stephen C. Graham[1]*

[1]Department of Pathology, University of Cambridge, Cambridge, UK

[2]Cambridge Institute for Medical Research, University of Cambridge, Cambridge, UK

[3]European Molecular Biology Laboratory (EMBL) Hamburg Site, Hamburg, Germany

[a]Current address: EG427 Pinpoint Gene Therapy, Paris, France

[b]Current address: The Francis Crick Institute, London, UK

***Corresponding author:** scg34@cam.ac.uk

This Supporting Information file contains: Figures S1 to S6, Tables S1 to S3, and Data S1.

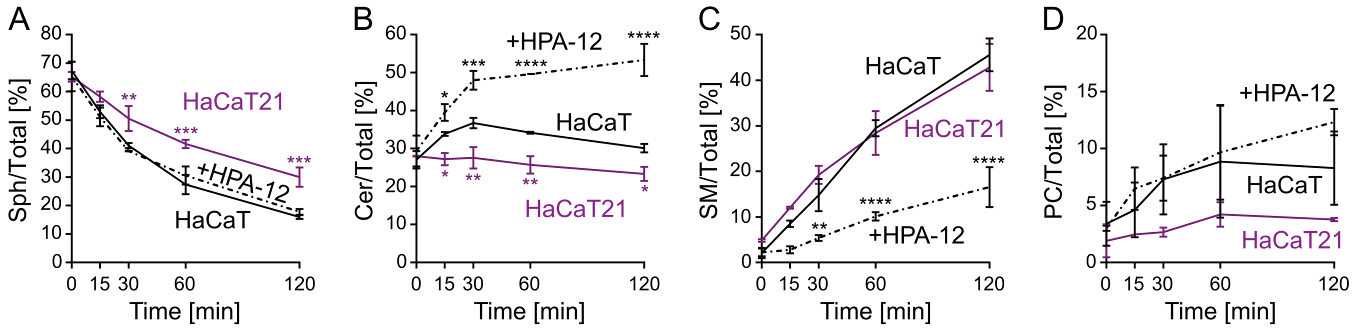

Figure S1. HSV-1 pUL21 alters the rate of sphingosine metabolism in cultured cells. Quantitation of the alkyne- (**A**) sphingosine (Sph), (**B**) ceramide (Cer), (**C**) sphingomyelin (SM) and (**D**) phosphatidylcholine (PC) intensities as percentage fraction of total signal. The data represents two independent experiments (mean ± SD). Data points are labelled if significantly different to parental HaCaT cells: *, $p < 0.05$; **, $p < 0.01$; ***, $p < 0.001$; ****, $p < 0.0001$ (two-way ANOVA with Dunnett's multiple comparisons test).

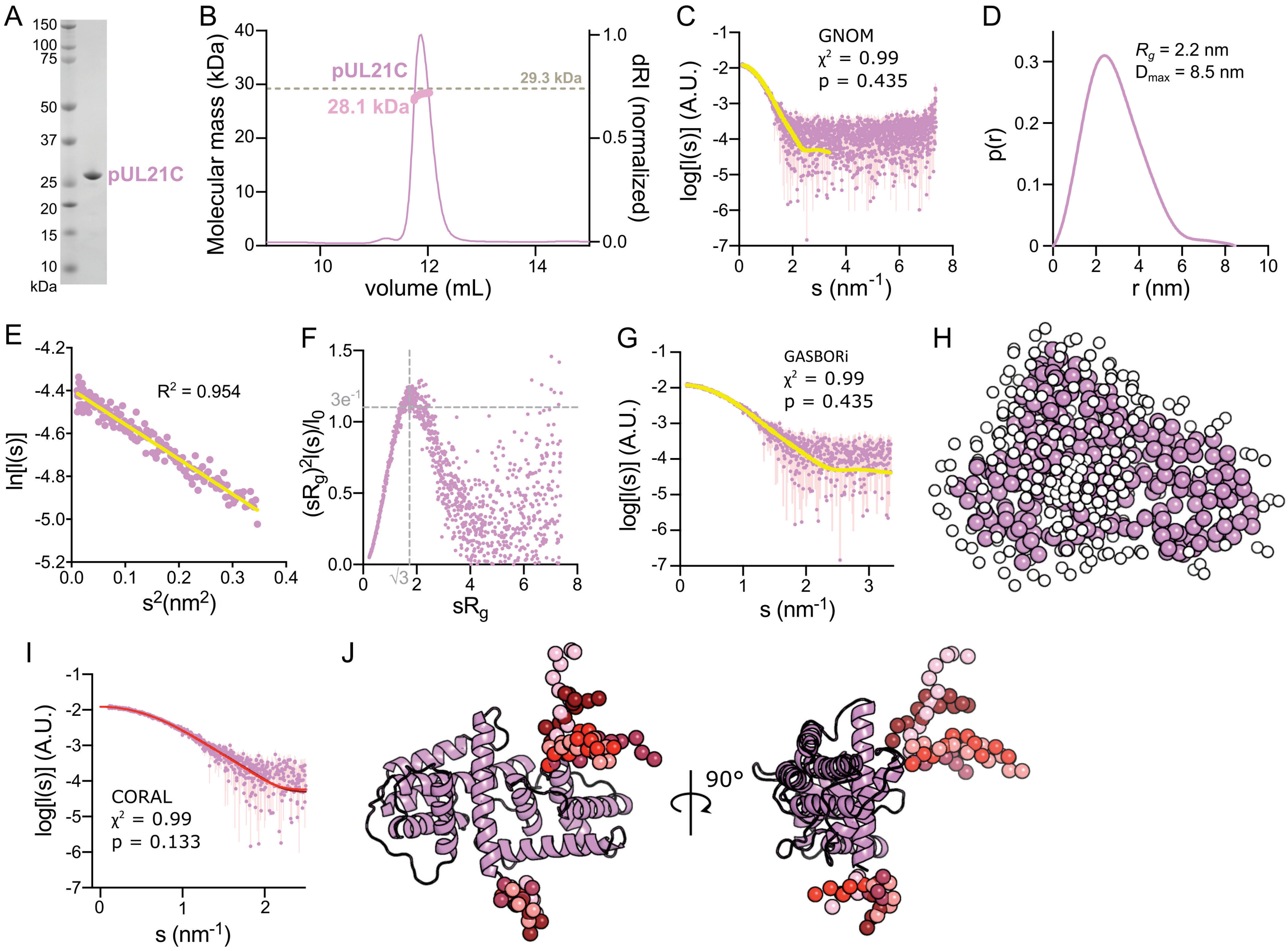

Figure S2. pUL21C is a monomeric globular protein. (**A**) SDS-PAGE of purified H6-pUL21C. Molecular weight markers are shown. (**B**) SEC-MALS elution profile (normalized differential refractive index, dRI) of H_6-pUL21C (dark pink, thin). The weight-averaged molecular mass (light pink, thick) is shown across the elution peak. The expected molecular mass for monomeric H_6-pUL21C is shown as a dotted horizontal line. (**C**) SAXS profile for H_6-pUL21C. The reciprocal-space fit of the *p(r)* profile to the SAXS data is shown as a yellow line. χ^2, fit quality; p, Correlation Map (CorMap) probability of systematic deviations between the model fit and the scattering data (93). (**D**) The real-space distance distribution function, $p(r)$, calculated from the SAXS profile. (**E**) The Guinier plot ($sR_g < 1.3$) is linear, representing an aggregate- and repulsion-free system. (**F**) Dimensionless Kratky plot. Grey dotted lines indicate the expected maximum of the plot for a compact protein ($sR_g = \sqrt{3}$, $(sR_g)^2I(s)/I(0) = 3e^{-1}$). (**G**) Fit of an *ab initio* dummy-residue model calculated using GASBOR to the SAXS profile. (**H**) GASBOR dummy-residue model. (**I**) Fit to SAXS profile of the five best pseudo-atomic models of H_6-pUL21C obtained by CORAL (continuous lines coloured in different shades of red, only topmost line is distinct because the lines overlay almost perfectly). χ^2 and CorMap p value are shown for one representative fit. (**J**) CORAL pseudo-atomic model of H_6-pUL21C. pUL21C (PDB ID 5ED7) (50) appears as violet ribbons and the regions modelled by CORAL are shown as spheres with colours corresponding to the fits shown in (**I**).

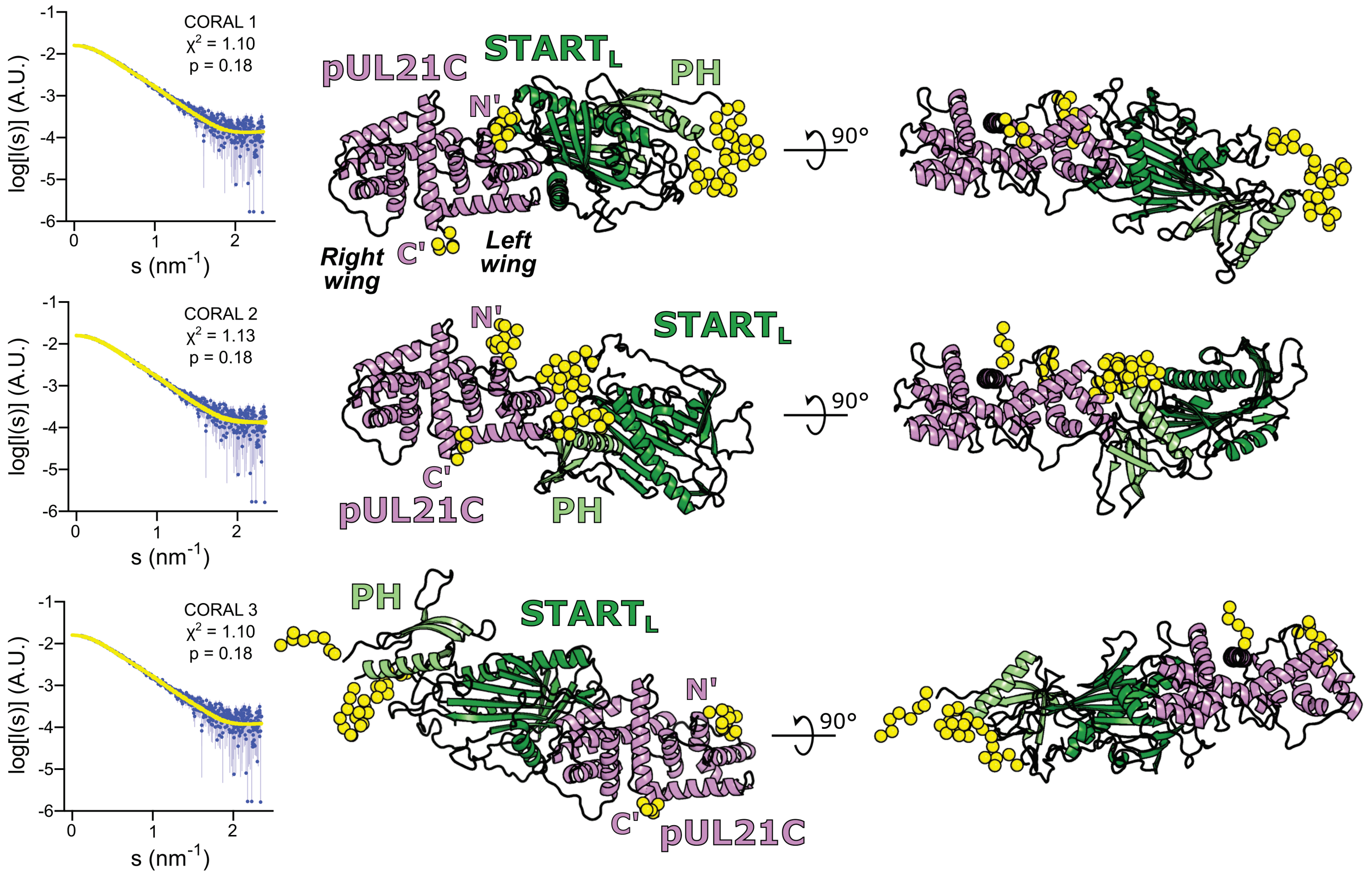

Figure S3. Pseudo-atomic models of the H_6-miniCERT$_L$:H_6-pUL21C complex. The best three pseudo-atomic models of the H_6-miniCERT$_L$:H_6-pUL21C complex were selected based on their fit to the SAXS data (Fig 3E, lowest χ^2). The fit of the computed scattering (yellow) to the H_6-miniCERT$_L$:H_6-pUL21C SAXS profile (blue) is shown for each model, as are χ^2 and CorMap p values. The pseudo-atomic models of the H_6-pUL21C (violet cartoon) and H_6-miniCERT$_L$ (PH, light green cartoon; STARTL, dark green cartoon) complex are shown in two orthogonal orientations. The left and right 'wings' of the dragonfly-like pUL21C fold (50) are labelled and the termini of the domain are marked. Regions absent from the crystal structures that were modelled by CORAL are shown as yellow spheres. *CORAL 1* (top) represents the model shown in Fig 3I, where the left wing of pUL21 binds miniCERT$_L$, whereas *CORAL 3* (bottom) represents the model shown in Fig 3J, where the right wing of pUL21 binds miniCERT$_L$. The fits to the scattering data for the *CORAL 1* and *CORAL 3* models, also shown in Fig 3I and 3J respectively, are included here for ease of comparison with the fit of the *CORAL 2* model.

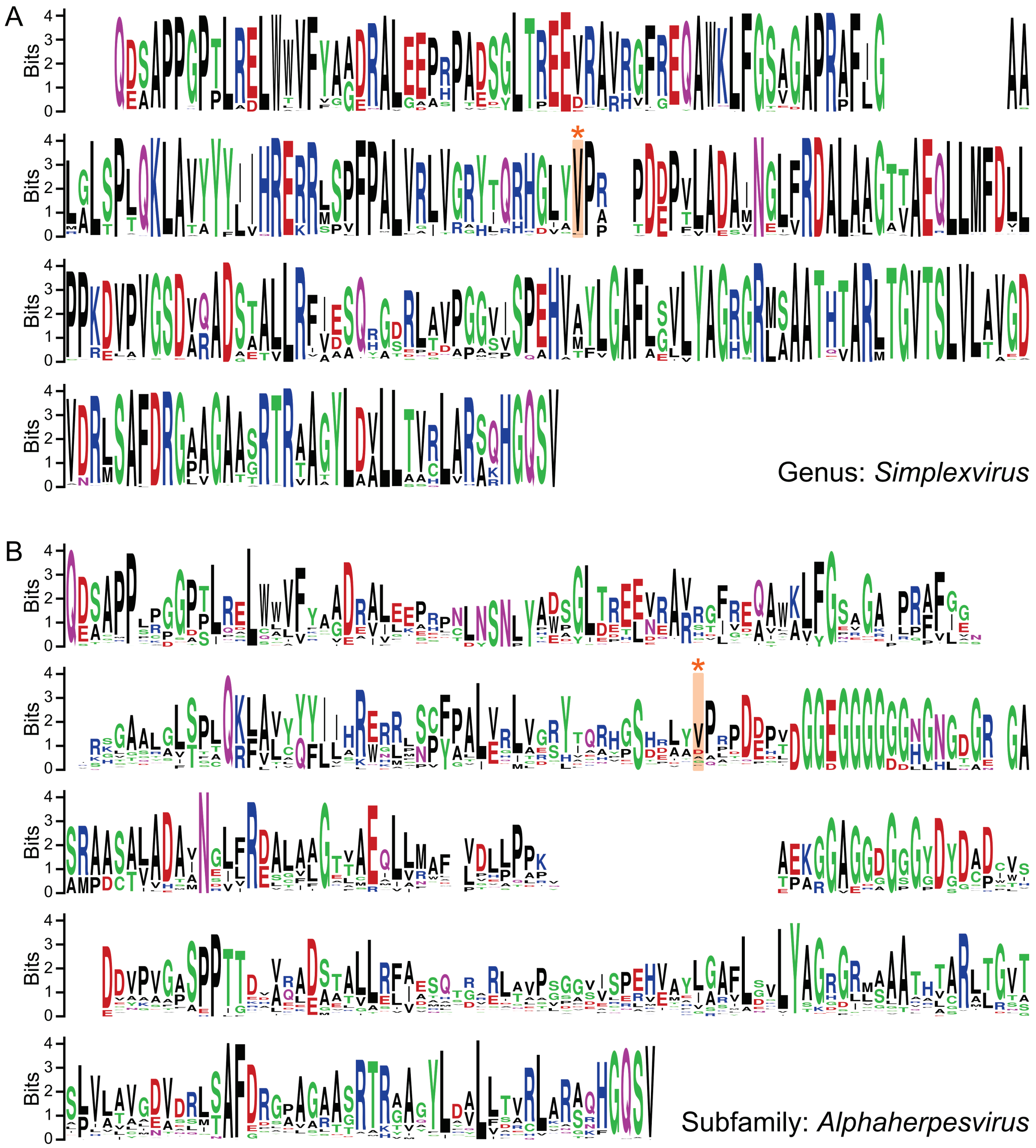

Figure S4. Sequence conservation of pUL21 C-terminal domain. The amino sequence of the pUL21 C-terminal domain (residues 275–535 of HSV-1) is shown at a WebLogo plot (106) with amino acid position heights scaled by information content. Logos were generated using pUL21 C-terminal domain sequences from across (**A**) the genus *Simplexvirus* (116 sequences aligned) or (**B**) the subfamily *Alphaherpesvirinae* (213 sequences aligned). HSV-1 pUL21 residue V382 is marked with an asterisk and highlighted in orange. Alignments are available as supporting information (Data S1).

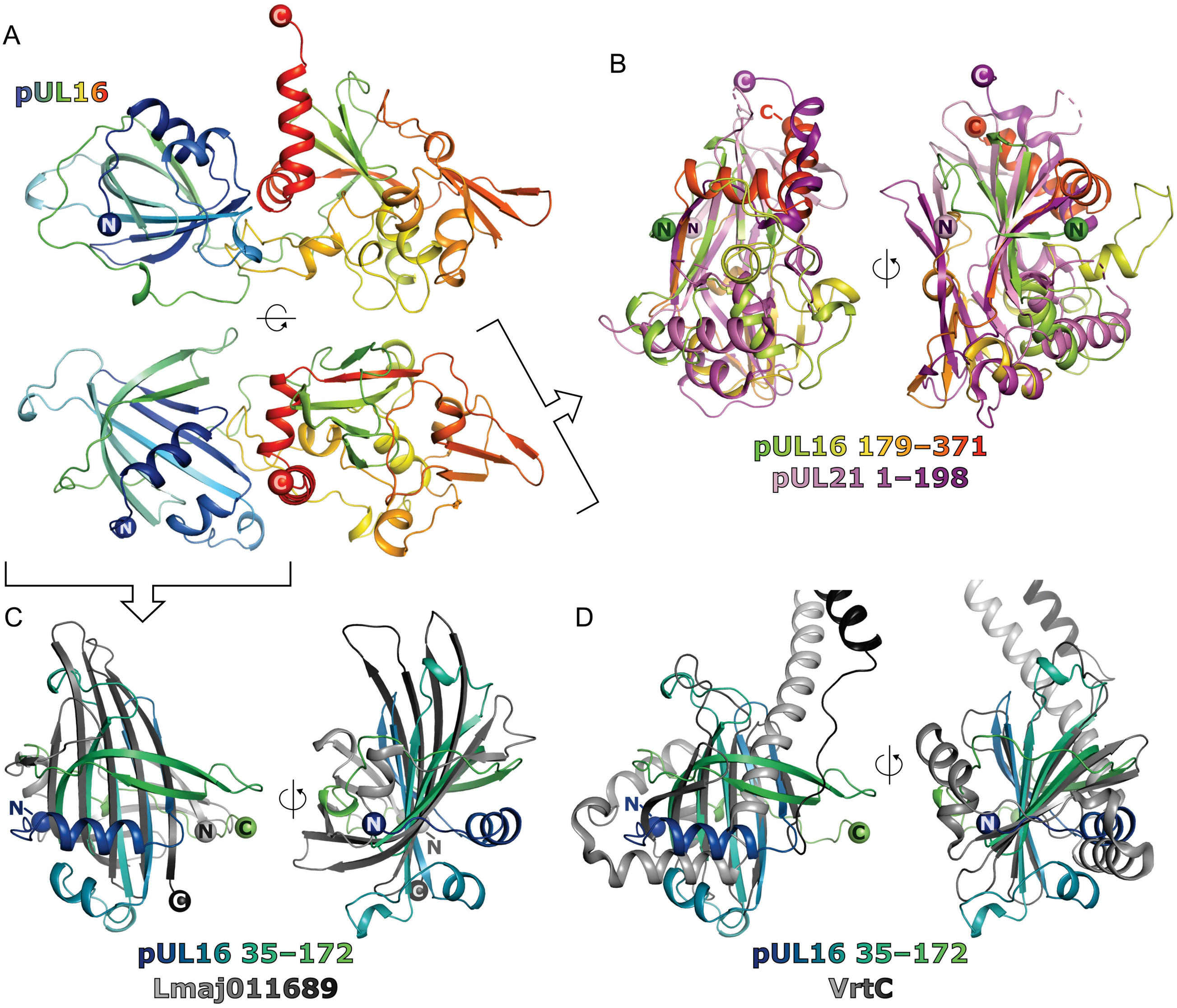

Figure S5. Predicted structure of HSV-1 pUL16. (**A**) AlphaFold2-Multimer model of pUL16 from HSV-1 (UniProt ID P10200), rainbow coloured from blue (N terminus) to red (C terminus). Residues 1–34 are omitted as they are predicted to be intrinsically unstructured. (**B**) Predicted structure of the pUL16 C-terminal domain superposed on the N-terminal domain of pUL21 (PDB ID 4U4H) (51), the highest scoring result from a DALI search (53) for structural homologues. (**C**) Predicted structure of the pUL16 N-terminal domain superposed on the hypothetical protein Lmaj011689 from leishmania major (PDB ID 1YQF), the highest scoring result from a DALI search for structural homologues. (**D**) Predicted structure of the pUL16 N-terminal domain superposed on the bile salt binding protein VrtC from *Vibrio parahaemolyticus* (PDB ID 5KEW) (79), the fourth highest scoring result from a DALI search for structural homologues.

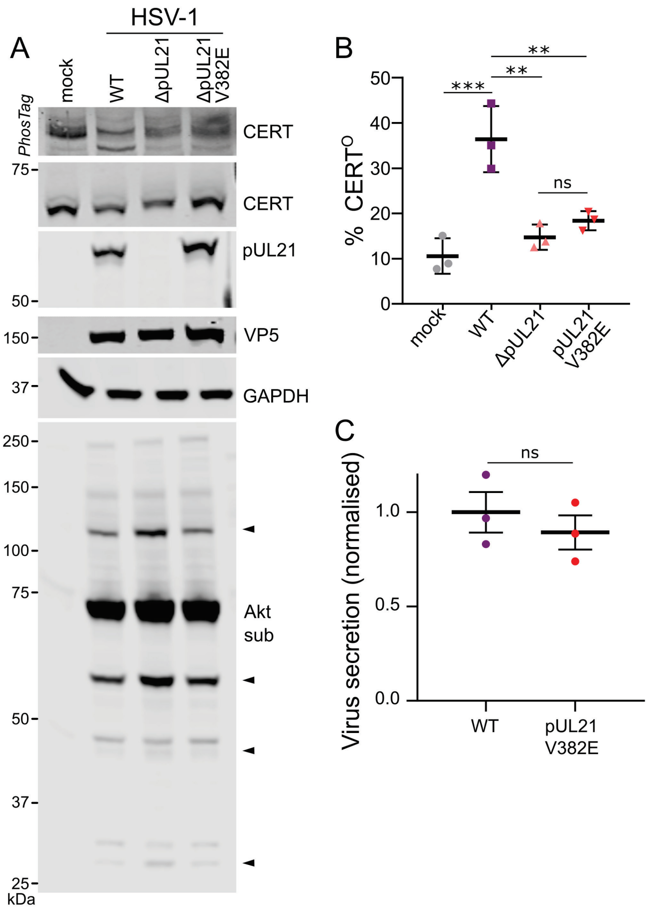

Figure S6. pUL21-mediated dephosphorylation of CERT in Vero cells does not impact HSV-1 secretion. (**A**) Vero cells were infected at MOI = 5 with wild-type (WT) HSV-1, HSV-1 lacking pUL21 expression (ΔpUL21), or a pUL21 point mutant virus ($pUL21^{V382E}$). Lysates were harvested at 16 hpi in the presence of phosphatase inhibitors and subjected to SDS-PAGE plus immunoblotting using the antibodies listed. Where indicated, the gel was supplemented with PhosTag reagent to enhance separation of CERT phosphoforms. The antibody recognising phosphorylated Akt substrates (Akt sub) illustrates activity of the HSV-1 kinase pUS3, several substrates of which are dephosphorylated in a pUL21-dependent manner (arrowheads) (18). VP5, infection control; GAPDH, loading control. (**B**) Quantitation of the CERT dephosphorylation level (ratio of $CERT^{O}$ to total CERT) in cells infected with WT or mutant HSV-1, as determined by densitometry. Results are presented as mean ± SD from three independent experiments. One-way ANOVA with Tukey's multiple comparisons test was used for the statistical analysis (ns, non-significant; **, $p < 0.01$; ***, $p < 0.001$). (**C**) Virus release into the culture supernatant from Vero cells infected with WT or mutant HSV-1 at MOI = 10. Samples were harvested at 12 hpi and virus infectivity in the cells versus the culture medium was measured by titration on Vero cells. The fold change in secretion of infectivity into the culture medium for $pUL21^{V382E}$ versus WT HSV-1 is shown as mean ± SD of one independent experiment performed in technical triplicate. The extent of virus secretion was compared using a two-tailed t-test (ns, non-significant).

Table S1. Isothermal titration calorimetry (ITC) of pUL21:CERT$_L$ interaction.

Syringe (μM)	Cell (μM)	K_D [nM]	ΔH [kcal/mol]	ΔG [kcal/mol]	-TΔS [kcal/mol]	N (stoichiometry)
StrepII-CERT$_L$^{P} [syringe] + *pUL21-H$_6$* [cell]						
328	28.9	1080.0	5.37	-8.15	-13.50	0.76
192	20.3	554.0	3.05	-8.54	-11.60	0.72
300	33.0	684.0	3.24	-8.41	-11.70	0.74
StrepII-CERT$_L$^{S132A} [syringe] + *pUL21-H$_6$* [cell]						
196	20.3	460.0	2.18	-8.65	-10.80	1.01
334	33.0	824.0	2.59	-8.30	-10.90	0.93
H$_6$-miniCERT$_L$ + *pUL21-H$_6$* [cell]						
376	29.9	720.0	-8.58	-8.38	0.19	0.91
376	29.9	866.0	-8.82	-8.27	0.55	0.88
330	37.6	897.0	-8.95	-8.25	0.70	0.83
372	28.9	760.0	-8.73	-8.35	0.38	0.97
195	20.3	879.0	-7.48	-8.26	-0.79	0.81
312	32.3	2130.0	-9.34	-7.73	1.61	0.91
H$_6$-miniCERT$_L$ [syringe] + *H$_6$-pUL21C* [cell]						
372	32.5	5500.0	-3.85	-7.18	-3.33	0.95
374	30.0	3150.0	-3.82	-7.51	-3.68	1.00
H$_6$-miniCERT$_L$ [syringe] + *pUL21^{V382E}-H$_6$* [cell]						
372	31.8	8390.0	-7.20	-6.93	0.28	1.22
196	19.4	8830.0	-7.16	-6.90	0.26	1.01
312	31.2	7040.0	-7.25	-7.02	0.23	1.25

Table S2. SAXS parameters.

	H_6-miniCERT$_L$	H_6-pUL21C	H_6-miniCERT$_L$: H_6-pUL21C
Data-collection parameters			
Radiation Source	Petra III (DESY, Hamburg, Germany)		
Beamline	EMBL P12		
Detector	DECTRIS Pilatus 6M		
X-ray wavelength (nm)	0.124		
Sample-to-detector distance (m)	3.0		
Temperature (°C)	20		
Exposure time (s), Data frames (#)	0.1, 47		
Measured protein concentrations (mg/mL)	1.73–6.91	0.68	NA
Injected protein concentration (mg/mL)	NA	NA	4.3
Measured *s*-range (nm^{-1})	0.07–7.219	0.08–7.37	0.05–7.36
Final working *s*-range (nm^{-1})	0.13–4.52	0.10–3.35	0.08–5.08
Structural parameters			
$I(0)$ (A.U.*) [from $p(r)$]	0.024 ± 1.5×10^{-5}	0.012 ± 6.4×10^{-5}	0.016 ± 5.9×10^{-5}
Real-space R_g (nm) [from $p(r)$]	2.7	2.2	3.5
$I(0)$ (A.U.*) (from Guinier)	0.024 ± 1.7×10^{-5}	0.012 ± 5.9×10^{-5}	0.016 ± 4.6×10^{-5}
R_g (nm) (from Guinier)	2.66	2.21	3.31
D_{max} (nm)	9.05	8.5	13.6
Porod volume estimate (Vp, nm^3)	70.5	11.7	85
Molecular-mass (M_r*) determination*			
M_r from Bayesian consensus (kDa)	46	32	56
M_r credibility interval (kDa)	43–47	30–33	47–58
Expected M_r from sequence (kDa)	44.9	29.3	73
Software employed			
Primary data reduction	*SASFLOW*		
Data processing	*PrimusQT/GNOM(5.0)*		
Ab initio analysis	*GASBOR(2.3.i)*		
Computation of model intensities	*CRYSOL(2.8.3)*		
Pseudoatomic modelling	*CORAL (1.1)*		
Small Angle Scattering Biological Data Bank			
SASBDB accession codes	*SASDNC7*	*SASDNB7*	*SASDND7*

*A.U. Arbitrary units

Table S3. Structures similar to the predicted N- and C-terminal domains of pUL16. Structural similarity searches were performed using DALI (53) using the predicted pUL16 N- and C-terminal domains (residues indicated). Only the top five results obtained when querying the PDB25 subset of the Protein Data Bank are shown for each domain.

	PDB ID	Chain	Z score	C^{α} rmsd	Aligned residues	Target residues	% a.a. identity[a]	Description	Reference
**pUL16 N-terminal domain (35–172)**	1YQF	A	5.2	3.4	66	182	6	Hypothetical protein LMAJ011689, similar to human p32 protein	Unpublished
	7D8G	A	4.9	3.0	81	174	9	Nucleotide phosphatase from *Staphylococcus aureus*	(107)
	3CBT	A	4.9	3.2	86	210	10	Phosphatase from *Streptomyces coelicolor*	Unpublished
	5KEW	B	4.8	4.1	71	132	6	Bile-salt binding protein VtrC	(79)
	4XZV	B	4.7	3.5	72	142	4	PRELI-like domain of SLMO1, part of the TRIAP1–SLMO1 mitochondrial phospholipid transport complex	(108)
**pUL16 N-terminal domain (179–371)**	4U4H	A	5.0	3.6	121	198	7	N-terminal domain of HSV-1 tegument protein pUL21	(51)
	5T87	G	2.9	2.6	54	75	4	Contact-dependent growth inhibition protein CdiA from *Cupriavidus taiwanensis*	(109)
	2DK7	A	2.8	3.4	55	73	7	WW domain of human transcription elongation regulator 1	Unpublished
	5B2O	A	2.1	4.0	67	1455	6	Cas9 nuclease from *Francisella novicida*	(110)
	5TXC	A	2.1	2.8	44	653	11	AtxE2 lasso peptide isopeptidase from *Asticcacaulis excentricus*	(111)

[a]Percent amino acid identity of aligned region

Data S1. Alignments of HSV-1 pUL21 C-terminal domain (residues 275–535) homologue amino acid sequences. Clustal formatted alignments are provided for simplexviruses (116 sequences; pUL21C_homologues_simplexvirus_COBALT.aln) and alphaherpesviruses (213 sequences; pUL21C_homologues_alphaherpesvirus_COBALT.aln).
